# Genome-Wide Characterization of the RNA Exosome Complex in Relation to Growth, Development, and Pathogenicity of Fusarium graminearum

**DOI:** 10.1128/spectrum.05058-22

**Published:** 2023-05-09

**Authors:** Yanping Yuan, Xuzhao Mao, Yakubu Saddeeq Abubakar, Wenhui Zheng, Zonghua Wang, Jie Zhou, Huawei Zheng

**Affiliations:** a Fujian Key Laboratory on Conservation and Sustainable Utilization of Marine Biodiversity, Fuzhou Institute of Oceanography, College of Geography and Oceanography, Minjiang University, Fuzhou, China; b Fujian Universities Key Laboratory for Plant-Microbe Interaction, College of Life Sciences, Fujian Agriculture and Forestry University, Fuzhou, China; c Department of Biochemistry, Ahmadu Bello University, Zaria, Nigeria; d College of Plant Protection, Fujian Agriculture and Forestry University, Fuzhou, Fujian, China; Universidade de Brasilia

**Keywords:** *Fusarium graminearum*, DON, RNA exosome complex, RNA processing, pathogenicity

## Abstract

The RNA exosome complex is a conserved, multisubunit RNase complex that contributes to the processing and degradation of RNAs in mammalian cells. However, the roles of the RNA exosome in phytopathogenic fungi and how it relates to fungal development and pathogenicity remain unclear. Herein, we identified 12 components of the RNA exosome in the wheat fungal pathogen Fusarium graminearum. Live-cell imaging showed that all the components of the RNA exosome complex are localized in the nucleus. *FgEXOSC1* and *FgEXOSCA* were successfully knocked out; they are both involved in the vegetative growth, sexual reproduction, and pathogenicity of F. graminearum. Moreover, deletion of *FgEXOSC1* resulted in abnormal toxisomes, decreased deoxynivalenol (DON) production, and downregulation of the expression levels of DON biosynthesis genes. The RNA-binding domain and N-terminal region of FgExosc1 are required for its normal localization and functions. Transcriptome sequencing (RNA-seq) showed that the disruption of *FgEXOSC1* resulted in differential expression of 3,439 genes. Genes involved in processing of noncoding RNA (ncRNA), rRNA and ncRNA metabolism, ribosome biogenesis, and ribonucleoprotein complex biogenesis were significantly upregulated. Furthermore, subcellular localization, green fluorescent protein (GFP) pulldown, and coimmunoprecipitation (co-IP) assays demonstrated that FgExosc1 associates with the other components of the RNA exosome to form the RNA exosome complex in F. graminearum. Deletion of *FgEXOSC1* and *FgEXOSCA* reduced the relative expression of some of the other subunits of the RNA exosome. Deletion of *FgEXOSC1* affected the localization of FgExosc4, FgExosc6, and FgExosc7. In summary, our study reveals that the RNA exosome is involved in vegetative growth, sexual reproduction, DON production, and pathogenicity of F. graminearum.

**IMPORTANCE** The RNA exosome complex is the most versatile RNA degradation machinery in eukaryotes. However, little is known about how this complex regulates the development and pathogenicity of plant-pathogenic fungi. In this study, we systematically identified 12 components of the RNA exosome complex in Fusarium head blight fungus Fusarium graminearum and first unveiled their subcellular localizations and established their biological functions in relation to the fungal development and pathogenesis. All the RNA exosome components are localized in the nucleus. FgExosc1 and FgExoscA are both required for the vegetative growth, sexual reproduction, DON production and pathogenicity in F. graminearum. FgExosc1 is involved in ncRNA processing, rRNA and ncRNA metabolism process, ribosome biogenesis and ribonucleoprotein complex biogenesis. FgExosc1 associates with the other components of RNA exosome complex and form the exosome complex in F. graminearum. Our study provides new insights into the role of the RNA exosome in regulating RNA metabolism, which is associated with fungal development and pathogenicity.

## INTRODUCTION

The RNA exosome complex is a conserved, multisubunit RNase complex that contributes to 3′-to-5′ RNA processing and degradation in eukaryotic cells (1–3). The RNA exosome complex is also present in archaea and bacteria (4). The RNA exosome of eukaryotes and archaea have a 9-subunit exosome core structure (Exo9) in common, which consists of a trimer cap and a hexamer ring (5). The trimer cap structure consists of three components, Csl4 (Exosc1), Rrp4 (Exosc2), and Rrp40 (Exosc3), while the hexameric ring structure consists of Rrp41 (Exosc4), Rrp46 (Exosc5), Mtr3 (Exosc6), Rrp42 (Exosc7), Rrp43 (Exosc8), and Rrp45 (Exosc9) (6, 7). The RNA exosome functions mainly in the nucleus and cytoplasm in eukaryotic cells. Two forms of exosome complex exist in the nucleus: Exo10^Rrp6^ and Exo11^Rrp44+Rrp6^ (5, 8). Exo10^Rrp6^ contains 10 components, with Exo9 and Rrp6 as the core subunits; Exo11^Rrp44+Rrp6^ contains 11 components, with Exo9, Rrp6 and Rrp44/Dis3 serving as the core components. In addition, Exo10^Rrp44/Dis3^ consists of the core subunits Exo9 and Rrp44/Dis3 in the cytoplasm. Contrary to what is observed in Saccharomyces cerevisiae, one component of the RNA exosome may contain multiple subunits in plant and mammalian cells. For example, Arabidopsis thaliana contains three RRP6-like (Rrp6L) proteins, namely, AtRrp6L1, AtRrp6L2 and AtRrp6L3 (9); also, the plant has two homologs of yeast Rrp45: AtRrp45a and AtCer7 (10).

The RNA exosome plays an important role in the degradation and processing of various RNAs and controls the quality of mRNA in response to cell differentiation and environmental changes (11). The specific recognition of the target transcript by RNA exosome is carried out by different cofactors that direct the RNA exosome to specific RNAs for processing or degradation (12, 13). Recently, researchers have found that mutation in genes that encode the various subunits of RNA exosome and their cofactors are associated with human diseases. Mutations in *EXOSC1*, *EXOSC2*, *EXOSC3*, *EXOSC5*, *EXOSC8*, and *EXOSC9* lead to a variety of neurodegenerative diseases (14–19). Knockdown of the RNA exosome complex cofactors *RBM7*, *SKIV2L*, and *TTC37* also leads to tissue-specific diseases with complex phenotypes (20, 21). In addition, it has been determined that the expression of the component of RNA exosome Dis3 is stage specific, and deletion of *DIS3* in *Drosophila* delays growth and induces the formation of melanoma, leading to death at the second-instar larval stage (22). Also in *Drosophila*, absence of Rrp6 and Rrp40 resulted in accumulation of small nucleolar RNA (snoRNA), while silencing of *dMTR3*, *dRRP6*, *dRRP41*, and *dRRP42* by RNA interference (RNAi) resulted in death of the adult insects (23). The loss of Exosc3 in β cells led to increased expression of transcription start site (TSS)-related antisense transcription RNA (xTSS-RNA) and accumulation of intracellular noncoding RNA (ncRNA), resulting in a high degree of somatic mutation (24). In Arabidopsis thaliana, mutations in Csl4 have no obvious phenotypic changes (25), and homozygous insertion mutations in transfer DNA (T-DNA) of AtRrp44, AtRrp45, and AtRrp46 are lethal (10, 26). Heterozygous mutations of AtRrp4, AtRrp41, and AtRrp42 lead to the death of female gametophytes during development (25, 27).

In yeast, all the RNA exosome complex genes were indispensable except *RRP6* (Exosc10) (28–32). In Ustilago maydis, posttranscriptional processing is the key factor for the regulation of pathogenicity (33–35). In Magnaporthe oryzae, the fungus-specific RNA-binding protein Rbp35 regulates the length of 3′ untranslated regions (UTRs) of transcripts having developmental and virulence-associated functions (36). MoCwf15, an essential splicing factor of the Prp19-associated component, regulates fungal growth and infection-related developments by modulating the intron splicing efficiency of a subset of genes in the rice blast fungus (37). In Botrytis cinerea, Nop53, a late-acting factor for 60S ribosomal subunit maturation, is crucial for the pathogen’s development and virulence (38). As an important part of the RNA quality control system, the RNA exosome complex is mainly involved in normal RNA degradation, abnormal transcription clearance, nuclear rRNA, and snoRNA and ncRNA processing in eukaryotes. In yeast and *Arabidopsis*, the RNA exosome is necessary for the maintenance of normal growth and development of the organism. In addition, mutations in subunits and cofactors of RNA exosome complex lead to a variety of human diseases. However, the functions of RNA exosome complex in plant-pathogenic fungi are still largely unknown.

Fusarium head blight (FHB) which is caused by Fusarium graminearum can result in serious economic losses, and mycotoxin contamination of wheat grains seriously threatens the health of humans and animals (39). F. graminearum can penetrate plant cells through the stomata, surface openings, or infection structures such as appressoria and infection cushions (40–42). The phytopathogen injects virulence factors into the host that interfere with the host immune defense factors, and this helps the fungus to spread in and colonize the host and eventually cause disease (43–45). Deoxynivalenol (DON) is one of the virulence factors of F. graminearum during infection (46). FgTri1 and FgTri4 are two cytochrome P450 oxygenases involved in early and late steps in trichothecene biosynthesis under trichothecene-inducing conditions in F. graminearum, and they are localized to spherical organelles, called toxisomes, that were presumed to be the site of trichothecene biosynthesis (39). *PRP4* encodes the only kinase among the spliceosome components; deletion of *FgPRP4* not only affects the intron splicing efficiency in over 60% of F. graminearum genes but also causes severe growth defects (47). Similarly, deletion of the RNA-binding protein FgRbp1 leads to reduced splicing efficiency in 47% of the F. graminearum intron-containing gene transcripts that are involved in various cellular processes, including vegetative growth, development, and virulence (48). Deletion mutants of FgSrp1, an SR (serine/arginine)-rich protein, rarely produce conidia, have reduced ascospore ejection and DON production, and cause only limited disease symptoms on wheat heads and corn silks (49). In addition, genetic mutations in specific selective splicing and A-to-I RNA editing during sexual reproduction in F. graminearum can cause defects in ascospore release (50–54). However, how RNA processing and degradation in pathogenic fungi regulate the mechanism of their unique infectivity and pathogenicity is still unclear.

In this study, we identified 12 candidate components of the RNA exosome complex in F. graminearum, and named them FgExosc1, FgExosc2, FgExosc3, FgExosc4, FgExosc5, FgExosc6, FgExosc7, FgExosc8, FgExosc9, FgExosc10, FgExosc11, FgExoscA, respectively. Among them, ExoscA is specifically distributed in some fungi. Furthermore, all the components of RNA exosome are localized in the nucleus in F. graminearum. FgExosc1 and FgExoscA are important for F. graminearum vegetative growth, development, and pathogenicity. The RBD and N-terminal region of FgExosc1 are both required for its normal localization and function. Transcriptome sequencing (RNA-seq) revealed that FgExosc1 is involved in ncRNA processing, rRNA and ncRNA metabolism, ribosome biogenesis and ribonucleoprotein complex biogenesis. Furthermore, GFP pulldown and coimmunoprecipitation (co-IP) experiments showed that FgExosc1 associates with the other components of the RNA exosome complex in F. graminearum.

## RESULTS

### Phylogenetic analysis of RNA exosome components.

To identify the components of RNA exosome in F. graminearum, we obtained the protein sequences of the components of RNA exosome in Saccharomyces cerevisiae and humans and identified 11 potential candidate proteins via BLASTp analysis, which we designated FgExosc1 (FGSG_13120), FgExosc2 (FGSG_09961), FgExosc3 (FGSG_08645), FgExosc4 (FGSG_01091), FgExosc5 (FGSG_09363), FgExosc6 (FGSG_04277), FgExosc7 (FGSG_10879), FgExosc8 (FGSG_08578). FgExosc9 (FGSG_05509), FgExosc10 (FGSG_06049), and FgExosc11 (FGSG_01184) (Table S1). In addition, we found a protein annotated as exosome-associated family (FGSG_08866) in the database FungiDB (https://fungidb.org), which we designated FgExoscA. Analyses of FgExoscA homologs revealed that it has no homologs in humans or S. cerevisiae, but it is present in a number of other filamentous fungi (Table S2), including Fusarium oxysporum, Magnaporthe oryzae, Sclerotinia sclerotiorum, and in some basidiomycete fungi (such as Ustilago maydis) as well as Aspergillus niger and Verticillium dahliae. These results suggest that ExoscA, a specific component of the RNA exosome, is widely distributed in fungi.

To further determine the architecture of RNA exosome components in F. graminearum, we analyzed the conserved domains and motifs of the 12 RNA exosome components. As shown in Fig. S1 in the supplemental material, FgExosc1, FgExosc2, and FgExosc3 have the typical conserved RNA binding domain (RBD) S1; FgExosc4, FgExosc5, FgExosc6, FgExosc7, FgExosc8 and FgExosc9 have the typical RNase_PH domain; FgExosc10 contained the PMC2NT domain at the N terminus and HRDC (helicase and RNase D C-terminal) domain at the C terminus. FgExosc11 contains a PilT N terminus (PIN) and a Vac domain. FgExoscA contains only one SAS10-UTP domain.

**FIG 1:**
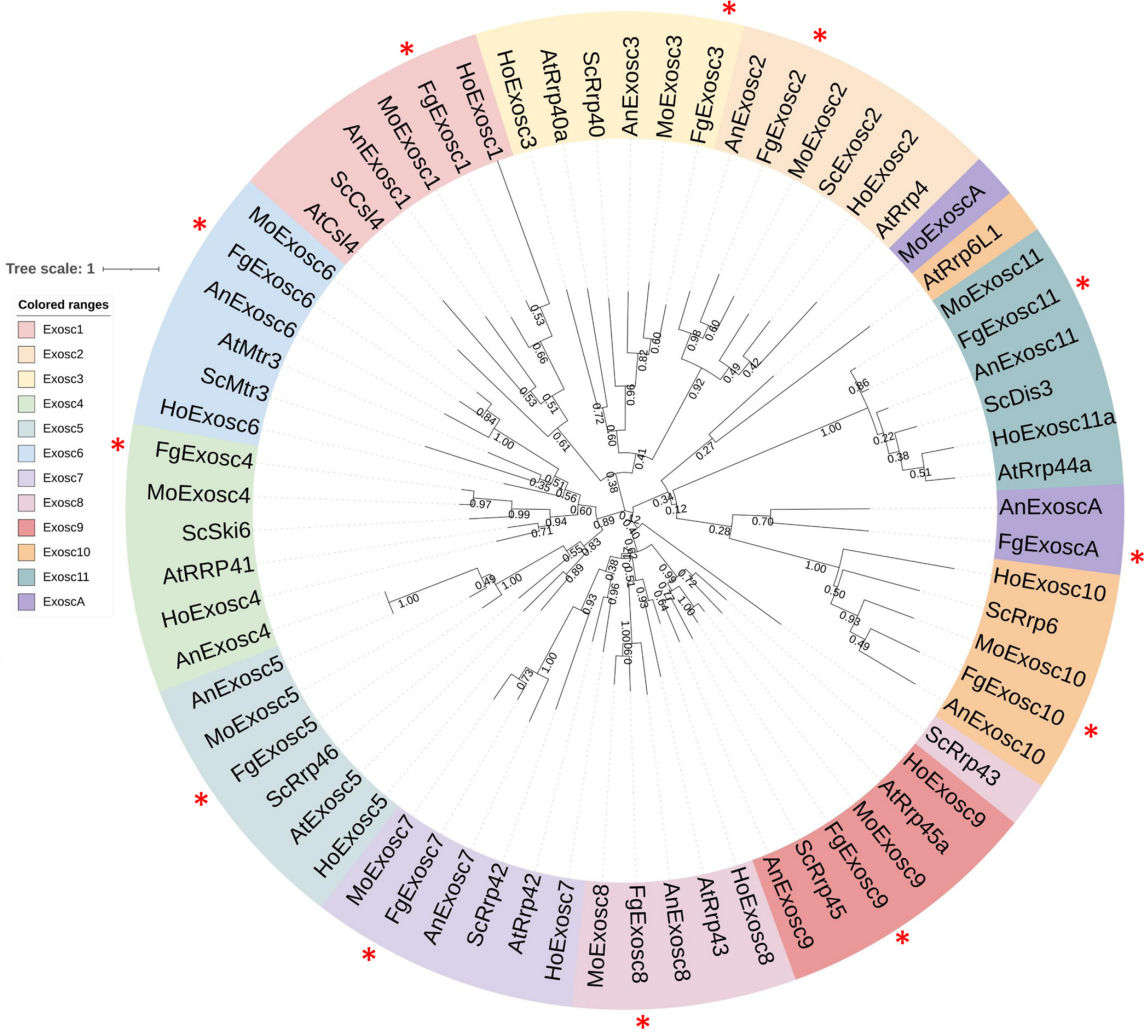
Phylogenetic analysis of subunits of RNA exosome complex. The phylogenetic tree was constructed with MEGAX software using the maximum-likelihood algorithm. The unrooted phylogenetic tree was plotted using the circular mode in the Interactive Tree Of Life (iTOL) v.6. The accession numbers of all the exosome components are provided in Table S2.

Further, we analyzed the phylogenetic relationship of RNA exosome components in F. graminearum in comparison with those in Saccharomyces cerevisiae, Magnaporthe oryzae, Aspergillus niger, Arabidopsis thaliana, and Homo sapiens, where we constructed a phylogenetic tree of the RNA exosome components in these species (Table S2). As shown in Fig. 1, the RNA exosome can be divided into three clades. Exosc1, -2, and -3 make up clade 1, Exosc4, -5, -6, and 10 make up clade 2, and Exosc7, -8, -9, -11, and -A make up clade 3, suggesting the divergence of the RNA exosome components in evolution. The evolutionary relationships of the components of RNA exosome have adaptive changes through evolution of species, which may lead to the different functions of those components in different species.

### RNA exosome subunits are localized to nucleolus in F. graminearum.

**FIG 2:**
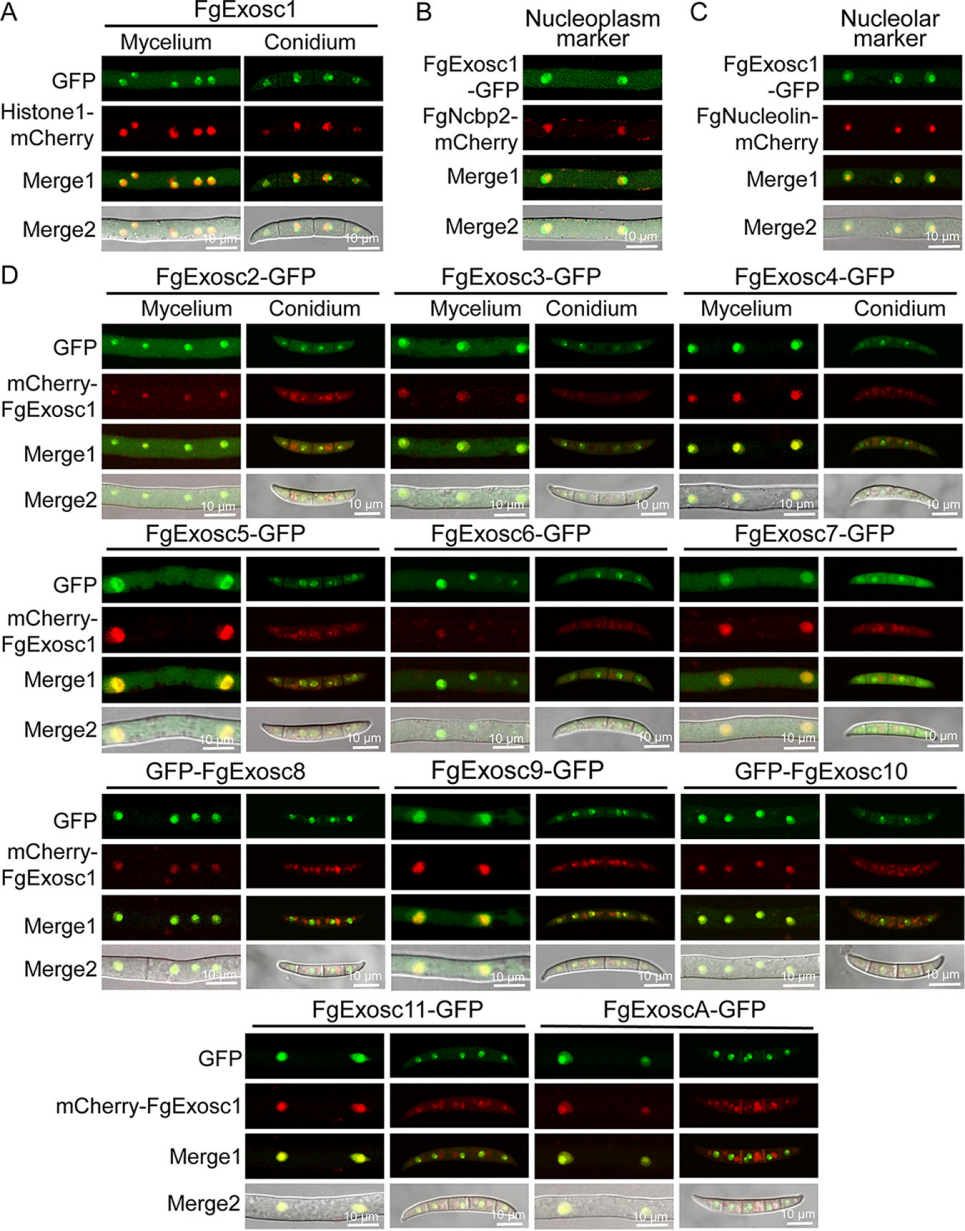
Subcellular localization of RNA exosome complex in mycelia and conidia of F. graminearum. (A) GFP-FgExosc1 colocalizes with the nuclear marker protein Histone1-mCherry in both mycelia and conidia of F. graminearum. Bar = 10 μm. (B and C) GFP-FgExosc1 colocalizes mainly with the nucleolus marker protein FgNucleolin-mCherry. (D) Colocalization of mCherry-FgExosc1 with the other components of the RNA exosome complex in the mycelia and conidia of F. graminearum.

To investigate the subcellular localization of RNA exosome in F. graminearum, we tagged each of the protein components with a green fluorescent protein (GFP), under the control of their native promoters. As shown in Fig. 2, the GFP signal of exosome complex displays distinct punctate signals along the cytoplasm, which we presumed to be nuclei. To check whether the GFP signals are really localized in the nuclei, we cotransformed the pGFP-FgExosc1 construct with a construct expressing the nuclear marker protein Histone1-mCherry into the protoplasts of the wild-type strain PH-1. We found that GFP-FgExosc1 colocalizes with the Histone1-mCherry signals in the nuclei of hyphae and conidia (Fig. 2A). To further confirm the precise localization of the RNA exosome within the nucleus, we cotransformed GFP-FgExosc1 with each of two constructs expressing the nucleolus marker FgNucleolin-mCherry and the nucleoplasm marker FgNcbp2-mCherry (a nuclear cap-binding protein) into the protoplasts of the wild-type strain PH-1 and observed their colocalizations by confocal microscopy. The results showed that GFP-FgExosc1 mainly colocalizes with FgNucleolin-mCherry (Fig. 2B and C), indicating that FgExosc1 is mainly localized to the nucleolus. Furthermore, we found that the other components of the RNA exosome are all colocalized with mCherry-FgExosc1 in the fungal mycelia and conidia (Fig. 2D). Taken together, these results indicate that all the components of the RNA exosome are mainly localized in the nucleolus of F. graminearum.

### FgExosc1 and FgExoscA are important for vegetative growth, conidiation, and sexual development of F. graminearum.

**FIG 3:**
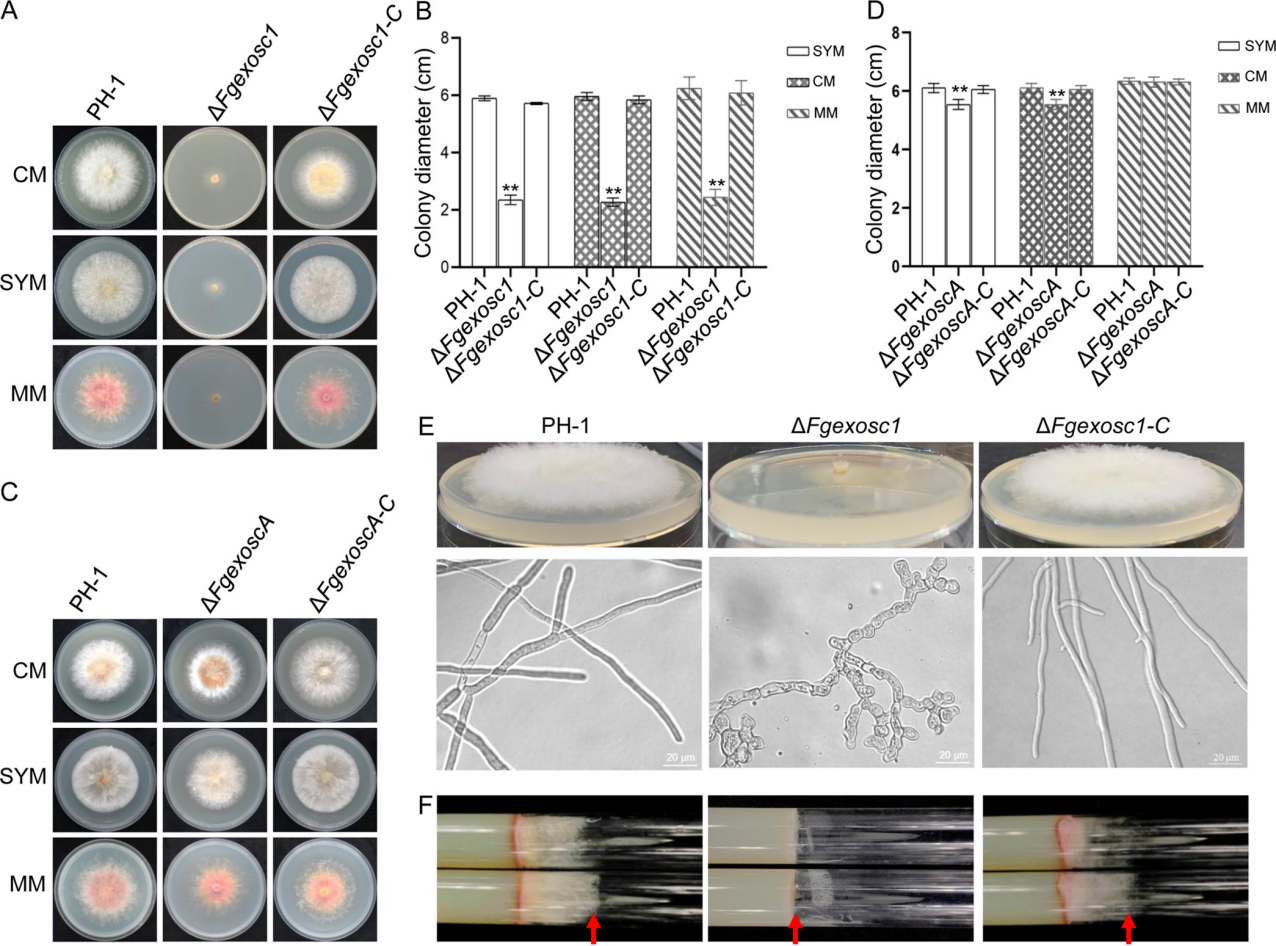
Colony morphologies and diameters of Δ*Fgexosc1* and Δ*FgexoscA* mutants. (A and B) Colony morphology and diameter of the Δ*Fgexosc1* mutant on CM, SYM, and MM after 3 days of incubation at 28°C. Error bars represent standard deviations from three replicates; a two-tailed Student’s *t* test was used for paired comparison of the diameters between PH-1 and the Δ*Fgexosc1* mutant (**, *P* < 0.01). (C and D) Colony morphology and diameter of the Δ*FgexoscA* mutant. **, *P* < 0.01. (E) Aerial hyphae and mycelial morphology of the Δ*Fgexosc1* mutant in CM agar plates and liquid CM, respectively. Bar = 20 μm. (F) Aerial hyphal morphology of the Δ*Fgexosc1* mutant in glass tubes containing CM agar.

To investigate the biological functions of RNA exosome in F. graminearum, *FgEXOSC1* and *FgEXOSCA* were successfully knocked out by homologous recombination (Fig. S2). However, we could not obtain mutants of the other 10 genes after several attempts. Phenotypic analysis of the Δ*Fgexosc1* mutant shows that its growth rate was severely decreased compared to that of wild-type strain PH-1 (Fig. 3A and B). On the other hand, deletion of *FgEXOSCA* has only a slight effect on the vegetative growth of F. graminearum (Fig. 3C and D). Moreover, as shown in Fig. 3E and F, the Δ*Fgexosc1* mutant displayed totally flattened mycelia on complete medium (CM) agar plates and in glass tubes containing CM agar. In addition, the Δ*Fgexosc1* mutant displayed aberrant mycelia with swelling and vacuolization in liquid CM compared to wild-type strain PH-1 (Fig. 3E). These results suggest that FgExosc1 is critical for vegetative growth and aerial hyphal development in F. graminearum. We performed gene complementation by reintroducing *FgEXOSC1* and *FgEXOSCA* genes (along with their respective native promoters) into the protoplasts of the Δ*Fgexosc1* and Δ*FgexoscA* mutants, respectively. We succeeded in generating the complemented Δ*Fgexosc1-C* and Δ*FgexoscA-C* strains, respectively. Consistently, Δ*Fgexosc1-C* and Δ*FgexoscA-C* strains were both found to have the phenotypic defects observed in all the mutants restored to the levels observed in the wild type.

**FIG 4:**
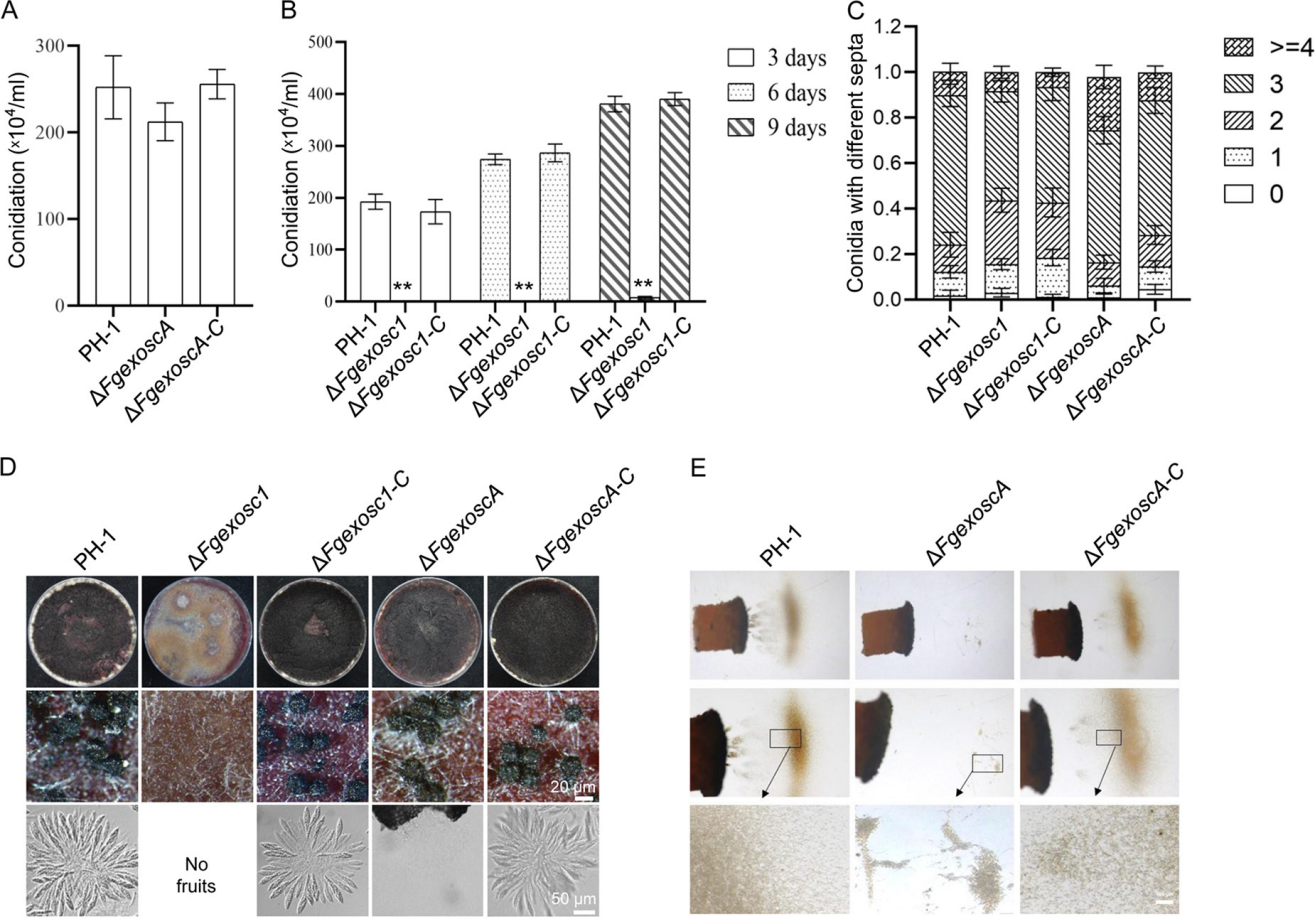
Roles of FgExosc1 and FgExoscA in asexual and sexual reproduction. (A) The number of conidia produced by PH-1 and the Δ*FgexoscA* and Δ*FgexoscA*-*C* strains on CMC medium for 3 days. (B) The number of conidia produced by PH-1 and the Δ*Fgexosc1* and Δ*Fgexosc1*-*C* strains on CMC medium for 3, 6, and 9 days. A two-tailed Student’s *t* test was used for paired comparison of conidiation between PH-1 and the Δ*Fgexosc1* strain (**, *P* < 0.01). (C) Analysis of the number of septa per conidium in the various strains. About 100 conidia from each of the indicated strains were counted in each experiment. (D) Perithecia and ascospores formed by PH-1 and the Δ*Fgexosc1*, Δ*FgexoscA*, Δ*Fgexosc1*-*C*, and Δ*FgexoscA-C* strains. Bar = 20 μm. The Δ*Fgexosc1* strain failed to form any perithecium. Bar = 50 μm. (E) Ascospores released by PH-1 and the Δ*FgexoscA* and Δ*FgexoscA-C* strains. Bar = 100 μm.

The conidia produced by F. graminearum are known to be the main inocula infecting flowering wheat heads (55). To characterize the role of the RNA exosome in conidium production by F. graminearum, wild-type strain PH-1, the Δ*Fgexosc1* and Δ*FgexoscA* mutants, and the complemented Δ*Fgexosc1-C* and Δ*FgexoscA-C* strains were inoculated into liquid carboxymethyl cellulose (CMC) medium. As shown in Fig. 4A and B, the conidiation of the Δ*Fgexosc1* mutant is clearly reduced in comparison to that produced by wild-type strain PH-1, while the Δ*FgexoscA* mutant had no significant difference. In fact, at 6 days postinoculation in CMC medium, the Δ*Fgexosc1* mutant failed to produce any conidia. However, very few conidia were recorded when the incubation time of the mutant culture was extended to 9 days. The number of septa per conidium in the Δ*Fgexosc1* and Δ*FgexoscA* mutants was similar to that observed in the wild-type strain conidia (Fig. 4C). In addition, we found that the Δ*Fgexosc1* mutant lost its ability to form perithecia (Fig. 4D). Although the Δ*FgexoscA* mutant produced perithecia normally, the number of ascospores formed was significantly reduced compared to that of wild-type strain PH-1 (Fig. 4D and E). These results indicate that FgExosc1 is required for conidiation and sexual development of F. graminearum, while FgExoscA is involved only in sexual development.

### FgExosc1 and FgExoscA are important for pathogenicity and DON production.

**FIG 5:**
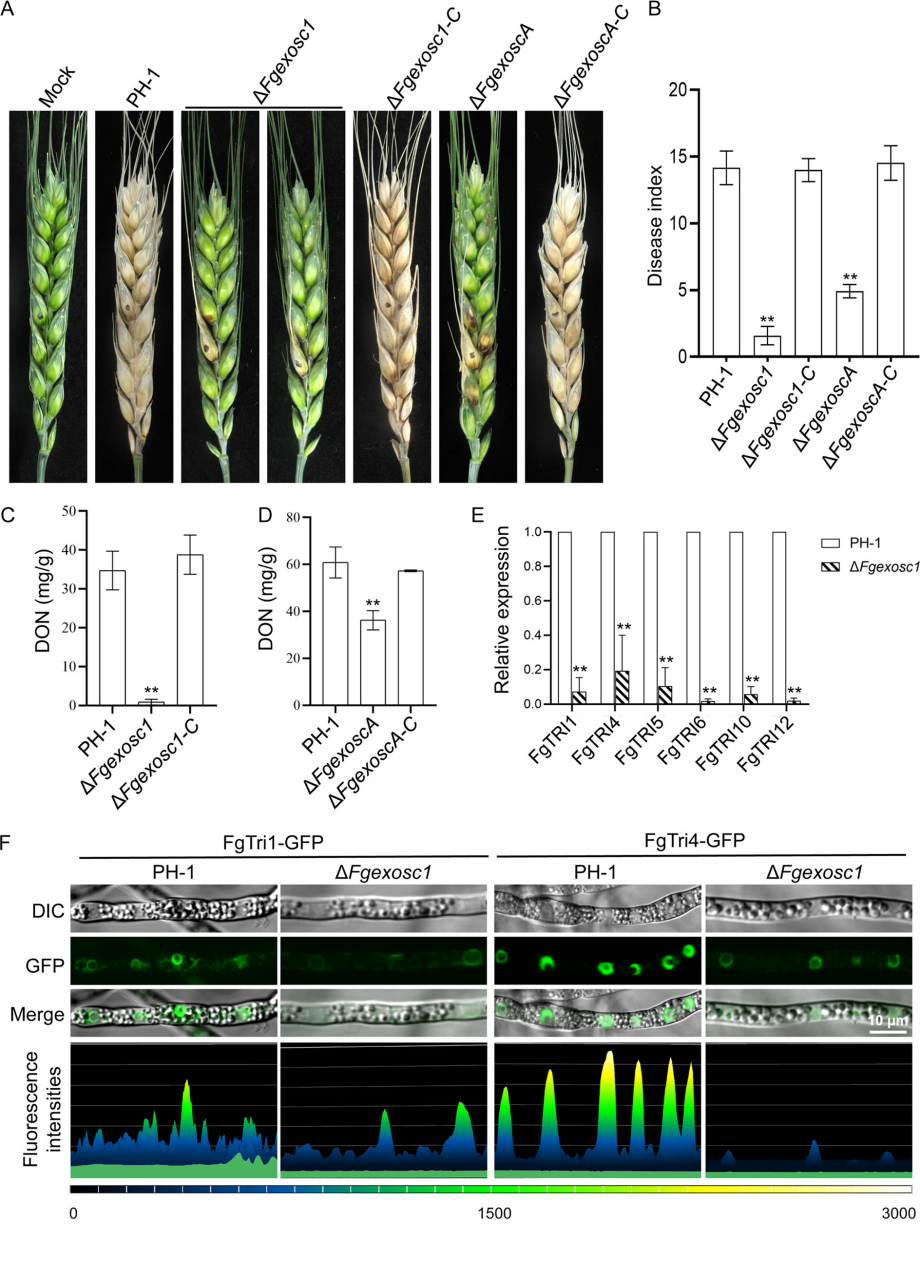
Roles of FgExosc1 and FgExoscA in pathogenicity and DON production of F. graminearum. (A) Deletion of *FgEXOSC1* and *FgEXOSCA* resulted in reduced pathogenicity of F. graminearum on flowering wheat heads; photographs were taken at 14 days postinoculation. (B) The disease index was based on the number of symptomatic spikelets. A two-tailed Student’s *t* test was used for paired comparison of the disease indices between PH-1 and the Δ*Fgexosc1* or Δ*FgexoscA* mutant (**, *P* < 0.01). (C and D) Deletion of *FgEXOSC1* and *FgEXOSCA* leads to reduced DON production in F. graminearum. **, *P* < 0.01. (E) Relative expression levels of DON biosynthesis genes (*FgTRI1*, *FgTRI4*, *FgTRI5*, *FgTRI6*, *FgTRI10*, and *FgTRI12*) in PH-1 and the Δ*Fgexosc1* mutant. **, *P* < 0.01. (F) Localization of FgTri1-GFP and FgTri4-GFP in PH-1 and the Δ*Fgexosc1* strain. Fluorescence intensities of FgTri1-GFP and FgTri4-GFP in PH-1 and the Δ*Fgexosc1* mutant are shown. Bar = 10 μm.

To characterize the role of FgExosc1 and FgExoscA in the pathogenicity of F. graminearum, wild-type strain PH-1 and the Δ*Fgexosc1*, Δ*FgexoscA*, Δ*Fgexosc1-C*, and Δ*FgexoscA-C* strains were inoculated on flowering wheat heads. As shown in Fig. 5A, the blight symptoms caused by Δ*Fgexosc1* and Δ*FgexoscA* mutants spread to the nearby spikelets at much lower rates than those caused by the wild type and the complemented strains under the same conditions. The average disease indices (number of diseased spikelets per head) of the Δ*Fgexosc1* and Δ*FgexoscA* mutants are 1.42 and 4.74, respectively, while the wild-type strain PH-1, Δ*Fgexosc1-C*, and Δ*FgexoscA-C* strains have indices of 13.44, 13.00, and 13.33, respectively (Fig. 5B). This suggests that FgExosc1 and FgExoscA are both important for the pathogenicity of F. graminearum.

Deoxynivalenol (DON) is one of the virulence factors of F. graminearum during infection (46). To verify whether the reduction in virulence of Δ*Fgexosc1* and Δ*FgexoscA* mutants is linked to reduction in DON production, we examined whether the *FgEXOSC1* and *FgEXOSCA* deletion mutants are deformed in DON production. To achieve this, we inoculated wild-type strain PH-1, the Δ*Fgexosc1* and Δ*FgexoscA* mutants, and the complemented Δ*Fgexosc1-C* and Δ*FgexoscA-C* strains into liquid trichothecene biosynthesis induction (TBI) medium. After 7 days of static culture at 28°C, DON production was determined. As shown in Fig. 5C, the amount of DON produced by Δ*Fgexosc1* mutant was significantly low (0.89 mg/g) compared to the amount produced by the wild type (34.74 mg/g). In addition, the amount of DON produced by the Δ*FgexoscA* strain was nearly half of that produced by the wild type (Fig. 5D). To further support these results, we assayed the expression levels of DON biosynthesis genes (*FgTRI1*, *FgTRI4*, *FgTRI5*, *FgTRI6*, *FgTRI10*, and *FgTRI12*) in the wild-type strain PH-1 and the Δ*Fgexosc1* mutant cultured in TBI medium. We found that the expression levels of these genes in the Δ*Fgexosc1* mutant were significantly downregulated compared to those in the wild type (Fig. 5E). Consistently, the fluorescence intensities of FgTri1-GFP- and FgTri4-GFP-labeled toxisomes in the Δ*Fgexosc1* mutant were significantly weaker than those observed in the wild type under same conditions, while the fluorescence of FgTri1-GFP- and FgTri4-GFP-labeled toxisomes were not significantly difference from those in wild-type strain PH-1 (Fig. 5F). In addition, the relative expression level of *FgTRI10* in the Δ*FgexoscA* mutant was significantly decreased compared to that in wild-type strain PH-1 (Fig. S3A), while the fluorescence intensities of FgTri1-GFP- and FgTri4-GFP-labeled toxisomes were not significantly different from those in wild-type strain PH-1 (Fig. S3B and C). Together, our data indicated that FgExosc1 and FgExoscA are both required for normal DON production and pathogenicity of F. graminearum.

### Functional characterization of the RBD and the N- and C-terminal regions of FgExosc1.

**FIG 6:**
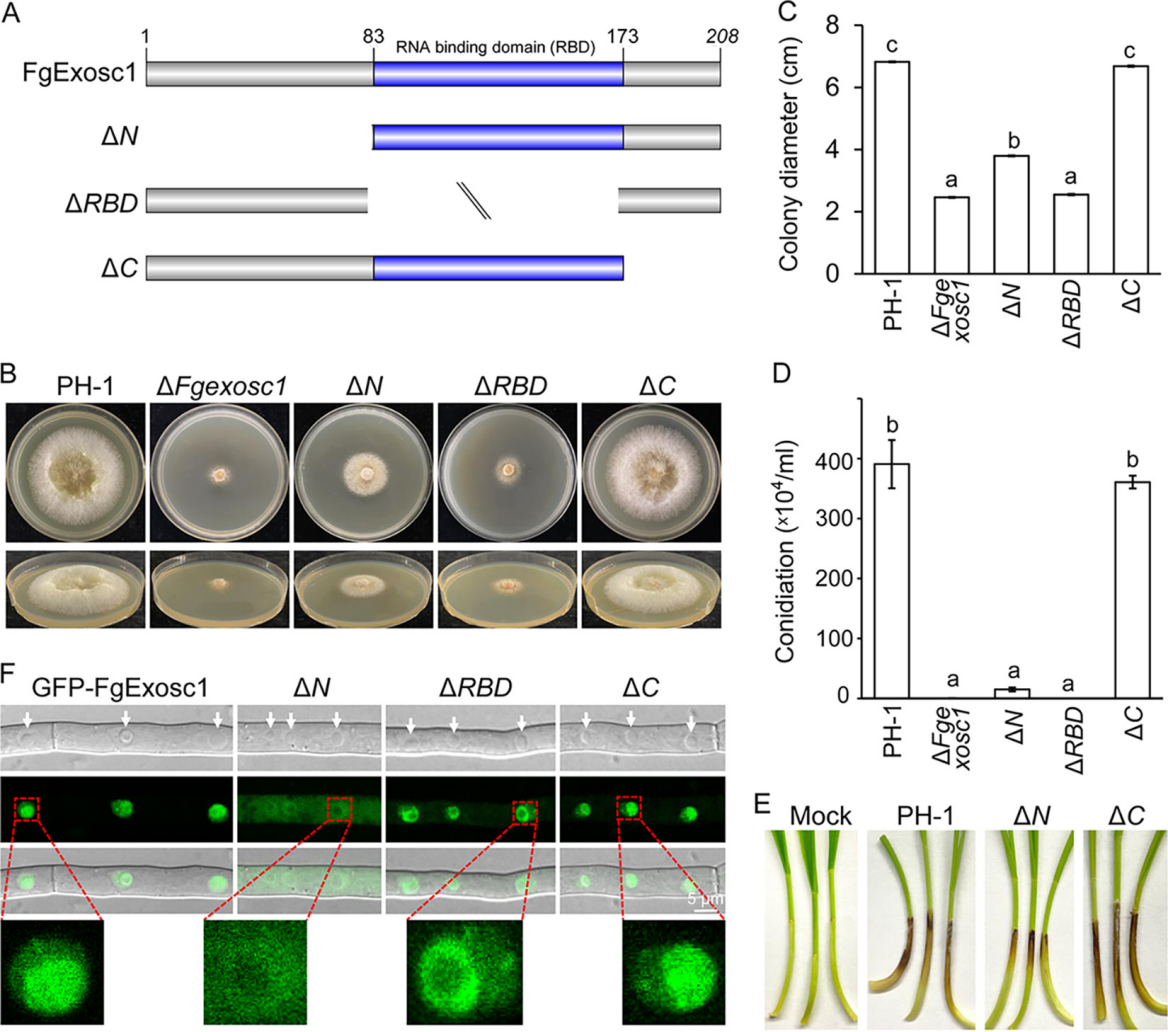
Functional characterization of the RBD and the N- and C-terminal regions of FgExosc1. (A) Schematic diagrams representing the deletions of the RBD and the N and C termini in FgExosc1. (B and C) Colony morphologies and diameters of PH-1 and the Δ*Fgexosc1*, Δ*N*, Δ*RBD*, and Δ*C* strains grown on CM for 3 days. Means and standard errors were calculated from three independent measurements; the same letter indicates a nonsignificant difference, as analyzed by Duncan’s test (*P* < 0.01). (D) The N-terminal region and RBD of FgExosc1 are both required for conidiation in F. graminearum. Means and standard errors were calculated from three independent measurements; the same letter indicates a nonsignificant difference, as analyzed by Duncan’s test (*P* < 0.01). (E) Pathogenicity of PH-1 and the Δ*N* and Δ*C* strains on wheat coleoptiles after 7 days of inoculation. The virulence of the Δ*N* mutant was significantly reduced. (F) The N-terminal region and RBD of FgExosc1 are both required for its proper localization in F. graminearum.

As mentioned above, FgExosc1 possesses an RNA-binding domain (RBD; amino acids [aa] 83 to 173) in F. graminearum (Fig. S1). To analyze the roles of this domain, we generated the GFP fusion constructs *GFP-FgEXOSC1*^Δ*RBD*^ (Δ*RBD*), *GFP-FgEXOSC1*^Δ*N*^ (Δ*N*), and *GFP-FgEXOSC1*^Δ*C*^ (Δ*C*) with deletions of the RBD (aa 83 to 173), N-terminal domain (aa 1 to 82), and C-terminal domain (aa 174 to 208), respectively (Fig. 6A). Next, these constructs were transformed into the protoplasts of the Δ*Fgexosc1* mutant to obtain the *GFP-Fgexosc1*^Δ*RBD*^ (Δ*RBD*), *GFP-Fgexosc1*^Δ*N*^ (Δ*N*), and GFP-*Fgexosc1*^Δ*C*^ (Δ*C*) mutants, respectively. Analyses of the phenotypes of these mutants revealed that the full-length RBD is required for the full functions of FgExosc1 in vegetative growth, conidiation, and pathogenicity, while the N-terminal region is only partially required (Fig. 6B to E and Fig. S4). However, the C-terminal region is dispensable for the functions of FgExosc1 in F. graminearum.

After deleting the N-terminal region of FgExosc1, we noticed that the GFP signal of GFP-FgExosc1^Δ*N*^ appeared diffuse in the cytoplasm and did not accumulate in the nucleolus (Fig. 6F), suggesting an important role of the N terminus in the nucleolus localization of FgExosc1. Interestingly, the GFP signal of GFP-Fgexosc1^Δ*RBD*^ displays a ring- or crescent-shaped appearance around the nucleolus but does not concentrate in the nucleolus (Fig. 6F), suggesting that deletion of the RBD of FgExosc1 disrupts its nucleolus localization. In addition, the localization of GFP-FgExosc1^Δ*C*^ (Δ*C*) was not significantly affected when its C-terminal region was deleted (Fig. 6F). From these results, we conclude that the N-terminal region and RBD of FgExosc1 are both required for the correct localization and functions of the protein, whereas the C-terminal region is dispensable not only for its function but also for its normal subcellular localization in F. graminearum.

### FgExosc1 is involved in processing and metabolism of various RNAs.

**FIG 7:**
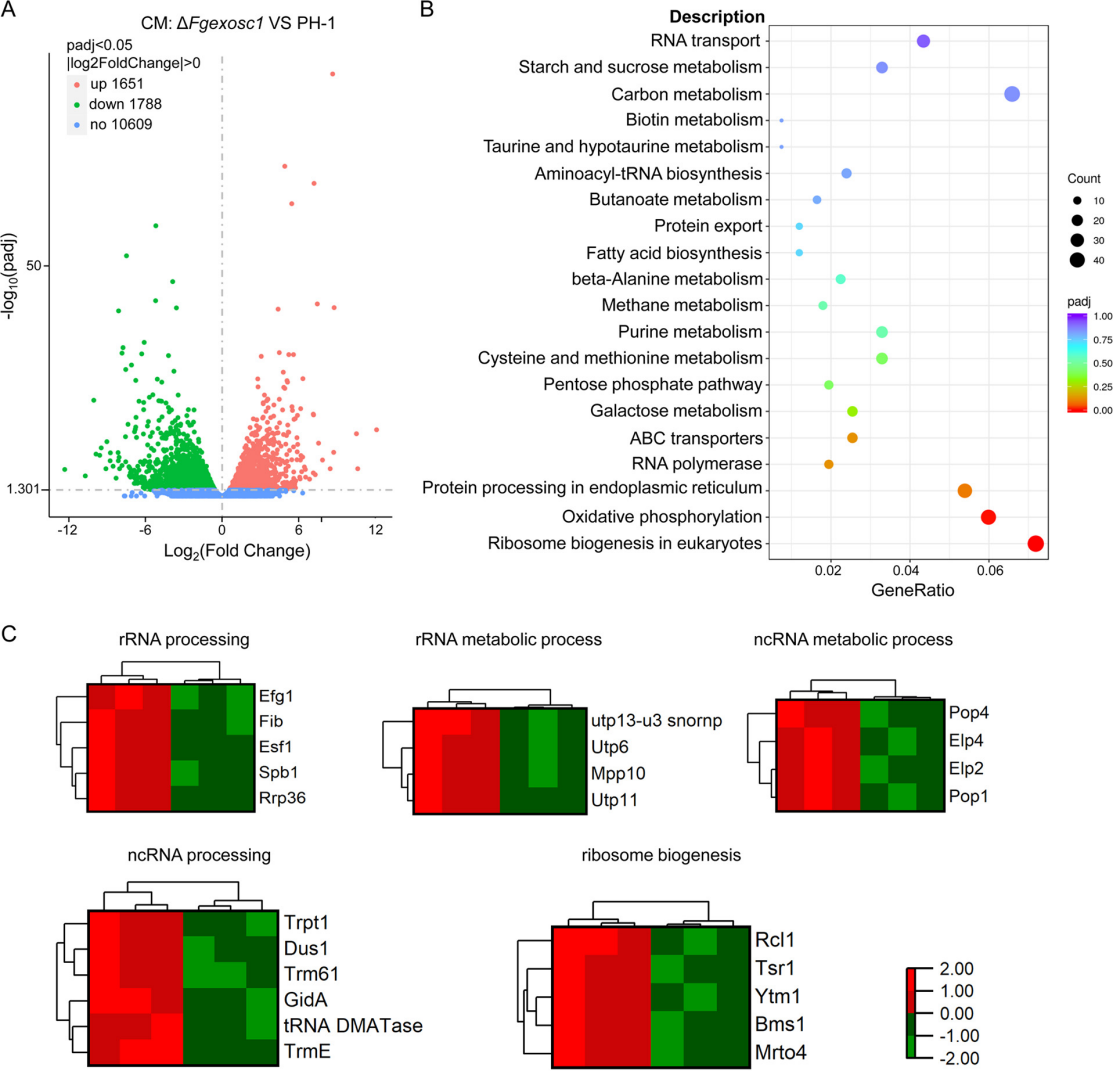
Assay for the role of FgExosc1 in transcriptional regulation. (A) Volcano plot of differentially expressed genes in Δ*Fgexosc1* and PH-1 strains cultured in CM. (B) KEGG enrichment analysis of the differentially expressed genes in Δ*Fgexosc1* and PH-1 strains. (C) Differentially expressed genes that are involved in rRNA processing, the rRNA metabolic process, the ncRNA metabolic process, ncRNA processing, and ribosome biogenesis in the Δ*Fgexosc1* mutant and PH-1.

The eukaryotic RNA exosome complex can process and degrade a variety of RNAs (13). We carried out RNA-seq for transcriptomic analyses of wild-type strain PH-1 and the Δ*Fgexosc1* mutants under nutrient-rich conditions. The results revealed that 1,788 genes were downregulated while 1,651 genes were upregulated in the Δ*Fgexosc1* mutant, compared to wild-type strain PH-1 (Fig. 7A). Based on GO (Gene Ontology) and KEGG (Kyoto Encyclopedia of Genes and Genomes) analyses, it was found that these differentially expressed genes are mainly related to small-molecule metabolism, ribosome biogenesis, rRNA metabolism and processing, and ncRNA processing (Fig. 7B). Genes that participate in rRNA metabolism and processing, ncRNA processing, and ribosome biogenesis are significantly upregulated (Fig. 7C). These results suggest that FgExosc1 plays an important role in RNA (ncRNA and rRNA) processing and metabolism in F. graminearum.

### FgExosc1 associates with the other components of the RNA exosome.

**FIG 8:**
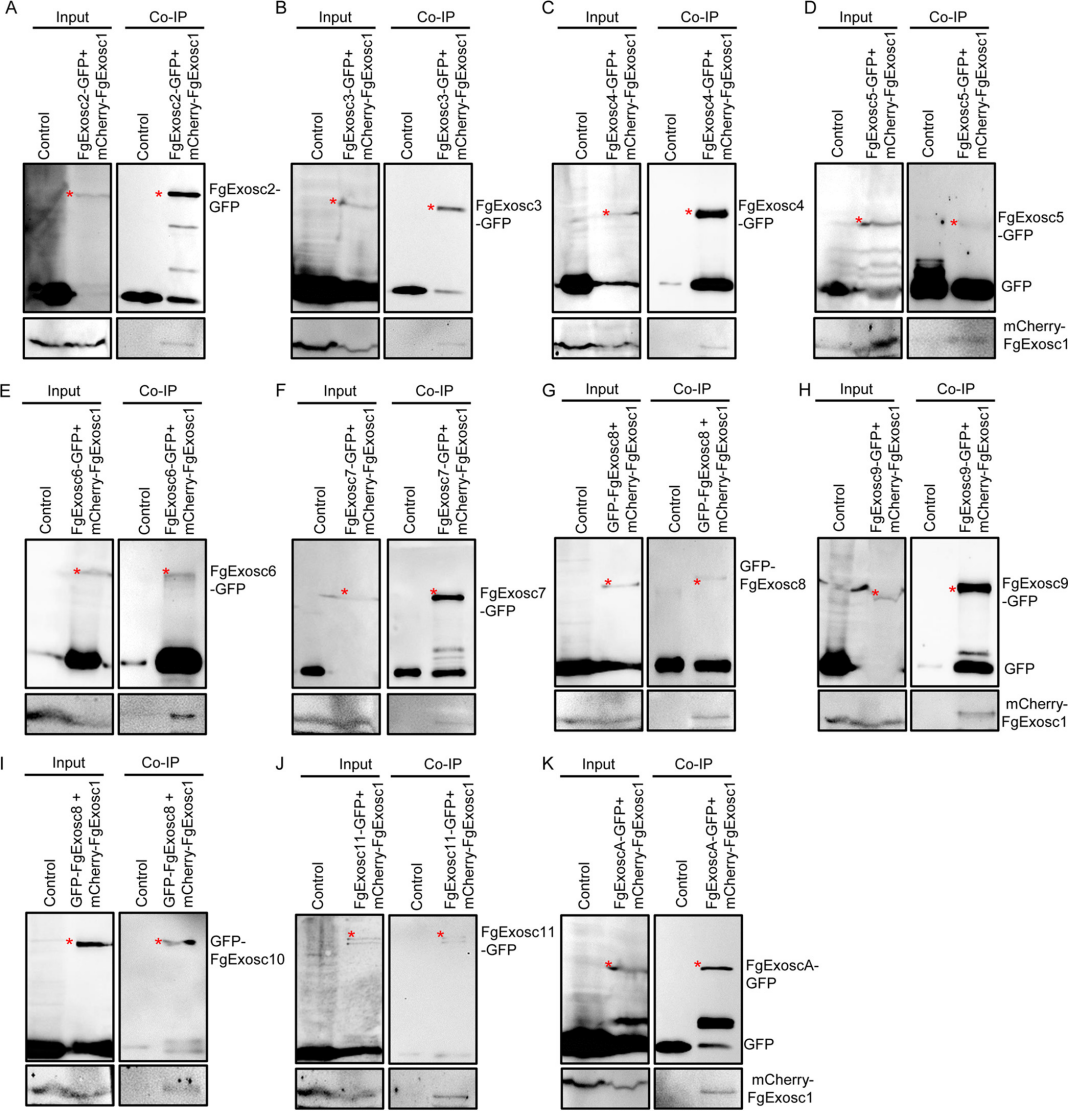
Analysis of FgExosc1 interaction with other components of RNA exosome by co-IP. (A to C) FgExosc1 interacts with FgExosc2, FgExosc3, and FgExosc4. (D to F) FgExosc1 interacts with FgExosc5, FgExosc6, and FgExosc7. (G to I) FgExosc1 interacts with FgExosc8, FgExosc9, and FgExosc10. (J and K) FgExosc1 interacts with FgExosc11 and FgExoscA.

**FIG 9:**
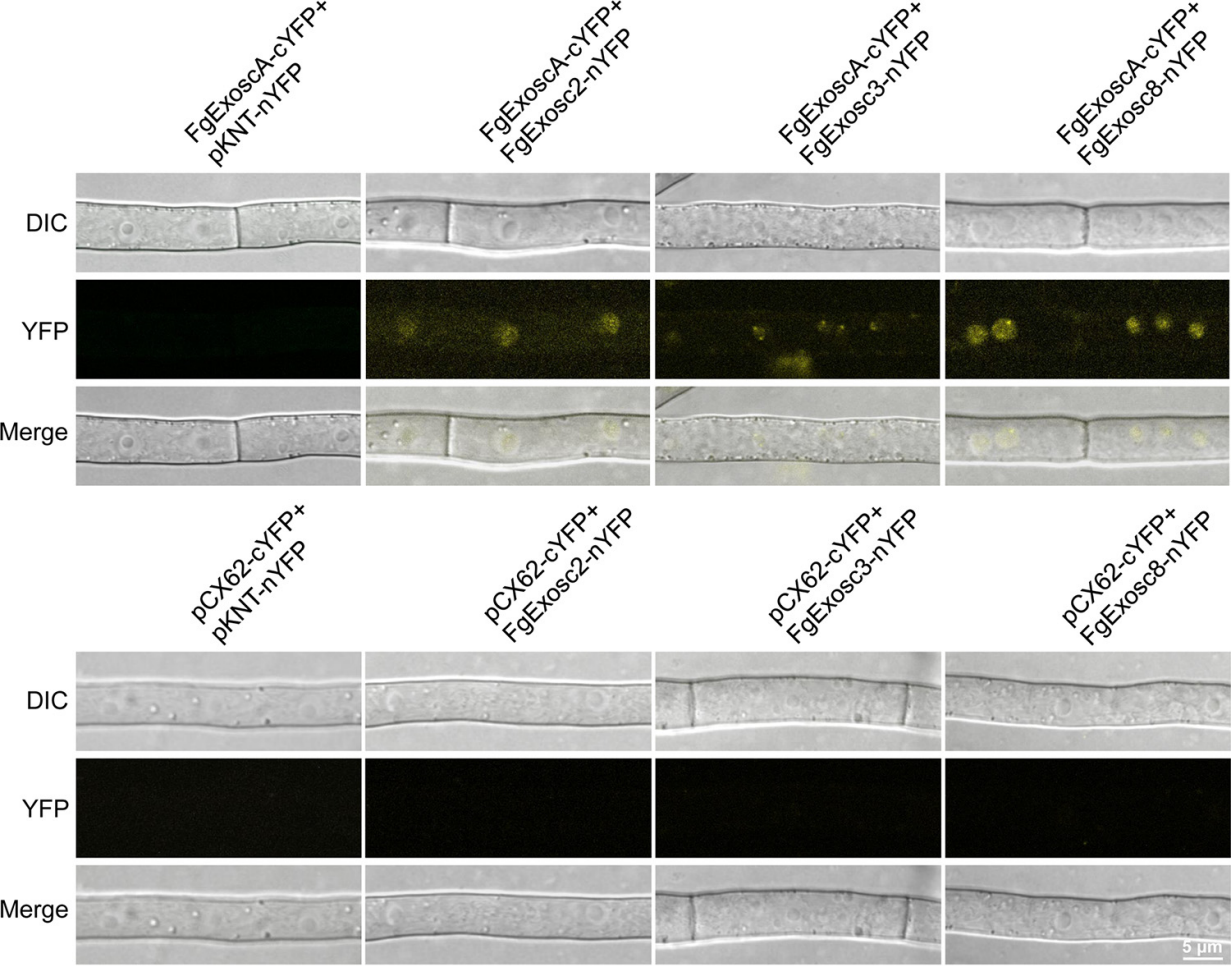
BiFC assay showing interaction of FgExoscA with other components of the RNA exosome. FgExoscA directly interacts with FgExosc2, FgExosc3, and FgExosc8.

The results presented above showed that FgExosc1 colocalizes with the other components of the RNA exosome in F. graminearum. To further establish the relationship between FgExosc1 and the other components of the RNA exosome, co-IP experiments were performed to search for possible interactions among the proteins. Consistently, the results showed that FgExosc1 interacts with FgExosc2, FgExosc3, FgExosc4, FgExosc5, FgExosc6, FgExosc7, FgExosc8, FgExosc9, FgExosc10, FgExosc11, and FgExoscA (Fig. 8). Moreover, the GFP-FgExosc1 strain was immunoprecipitated using a GFP-Trap kit to confirm the above interactions. The proteins bound to the GFP-Trap beads were eluted and identified by liquid chromatography-tandem mass spectrometry (LC-MS/MS). Again, FgExosc2, FgExosc3, FgExosc4, FgExosc5, FgExosc6, FgExosc7, FgExosc8, FgExosc9, FgExosc10, FgExosc11, and FgExoscA were identified in the pulldown assays of GFP-FgExosc1 (Table 1). For *in vivo* confirmation of these results, a bimolecular fluorescence complementation (BiFC) assay was performed to visualize the interactions in living cells. As shown in Fig. S5, FgExosc1 directly interacts with each of FgExosc2, FgExosc6, FgExosc7, and FgExoscA in the nucleus of F. graminearum. However, FgExosc1 does not directly interact with other RNA exosome components. Furthermore, BiFC was also used to check for possible interaction of FgExoscA with FgExosc2, FgExosc3, FgExosc4, FgExosc5, FgExosc6, FgExosc7, FgExosc8, FgExosc9, FgExosc10, and FgExosc11. The results showed that FgExoscA directly interacts with FgExosc1, FgExosc2, FgExosc3, and FgExosc8 *in vivo* (Fig. 9; FIG. S5, suggesting that FgExoscA directly associates with FgExosc1, FgExosc2, FgExosc3, and FgExosc8. Taken together, these results demonstrate that FgExosc1 associates with FgExosc2, FgExosc6, FgExosc7, FgExoscA, FgExosc3, FgExosc4, FgExosc5, FgExosc8, FgExosc9, FgExosc10, and FgExosc11 to form the RNA exosome complex by directly or indirectly interacting in F. graminearum.

**TABLE 1:** Numbers of peptides of the exosome complex identified by GFP-FgExosc1 pulldown

Component of RNA exosome complex	No. of peptides (unique) in replication:		
	1	2	3
FgExosc1 (FGSG_13120)	10	8	9
FgExosc2 (FGSG_09961)	36	8	12
FgExosc3 (FGSG_08645)	14	3	8
FgExosc4 (FGSG_09363)	12	4	4
FgExosc5 (FGSG_01091)	14	6	7
FgExosc6 (FGSG_04277)	17	3	6
FgExosc7 (FGSG_10879)	17	6	8
FgExosc8 (FGSG_08578)	18	7	8
FgExosc9 (FGSG_05509)	13	3	7
FgExosc10 (FGSG_06049)	1	0	0
FgExosc11 (FGSG_01184)	40	13	19
FgExoscA (FGSG_08866)	2	0	1

**FIG 10:**
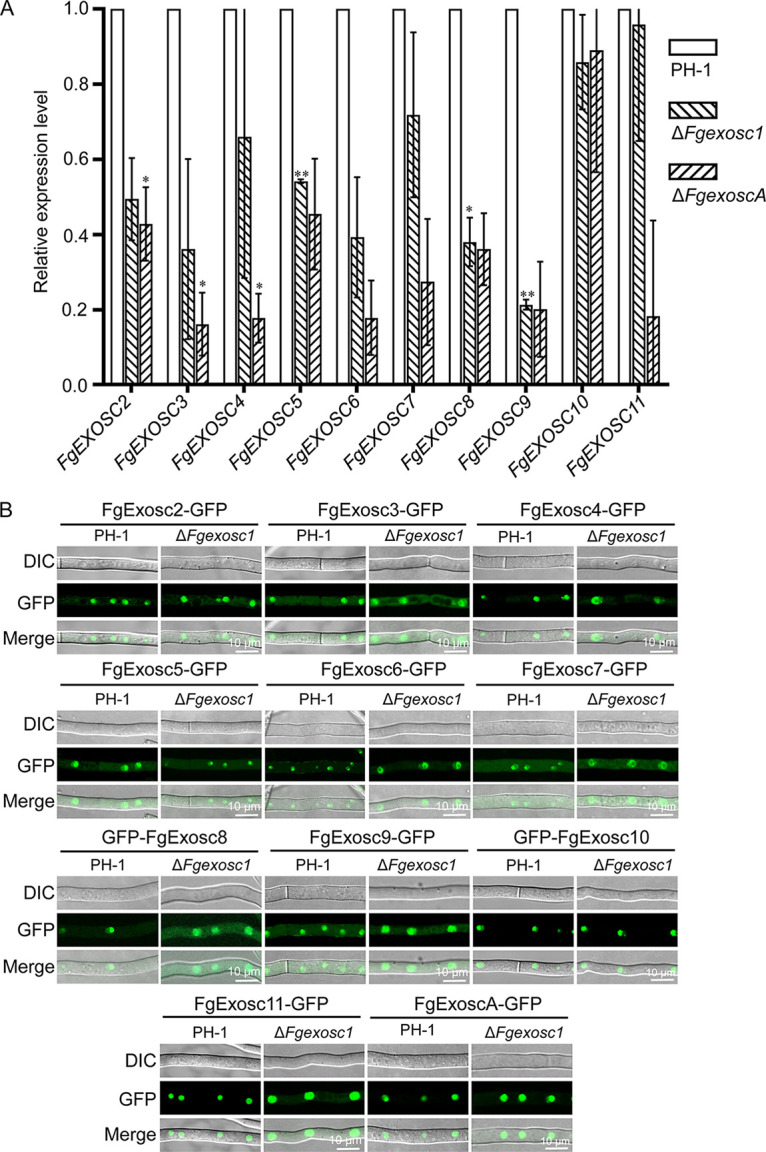
Effects of *FgEXOSC1* and *FgEXOSCA* deletions on the expression levels of the genes encoding the other components of RNA exosome complex in F. graminearum. (A) Deletion of *FgEXOSC1* caused downregulation of *FgEXOSC5*, *FgEXOSC8*, and *FgEXOSC9*; deletion of *FgEXOSCA* caused downregulation of *FgEXOSC2*, *FgEXOSC3*, and *FgEXOSC4*. *, *P* < 0.05; **, *P* < 0.01. (B) The deletion of *FgEXOSC1* affected the localizations of FgExosc4-GFP, FgExosc6-GFP, and FgExosc7-GFP in F. graminearum.

To investigate whether disruption of any one of the RNA exosome components could affect the homeostasis of other components, we first checked the expression levels of other subunits of the RNA exosome in Δ*Fgexosc1* and Δ*FgexoscA* mutants. As shown in Fig. 10A, deletion of *FgEXOSC1* caused downregulation of *FgEXOSC5*, *FgEXOSC8*, and *FgEXOSC9*, while the expression levels of *FgEXOSC2*, *FgEXOSC3*, *FgEXOSC4*, *FgEXOSC6*, *FgEXOSC7*, *FgEXOSC10*, and *FgEXOSC11* were not significantly affected. In addition, the expression levels of *FgEXOSC2*, *FgEXOSC3*, and *FgEXOSC4* in the Δ*FgexoscA* mutant were significantly downregulated compared to that in PH-1. These results suggest that loss of a single component of the RNA exosome may affect the homeostasis of other components in F. graminearum.

Next, we further studied whether the deletion of *FgEXOSC1* affects the subcellular localizations of the other components of the RNA exosome complex by checking the localizations of the other subunits in the Δ*Fgexosc1* mutant. FgExosc2-GFP, FgExosc3-GFP, FgExosc4-GFP, FgExosc5-GFP, FgExosc6-GFP, FgExosc7-GFP, GFP-FgExosc8, FgExosc9-GFP, GFP-FgExosc10, FgExosc11-GFP, and FgExoscA-GFP vectors were transferred into the protoplasts of Δ*Fgexosc1* mutant. As shown in Fig. 10B, the localizations of FgExosc2, FgExosc3, FgExosc5, FgExosc8, FgExosc9, FgExosc10, FgExosc11, and FgExoscA were not significantly altered in the absence of *FgEXOSC1* compared to the wild type. However, the localizations of FgExosc4, FgExosc6, and FgExosc7 were significantly different from those in the wild type; the signals of FgExosc4-GFP, FgExosc6-GFP, and FgExosc7GFP accumulated in the rings around the nucleolus in the Δ*Fgexosc1* mutant. Taken together, these results suggest that FgExosc1 is not necessary for the correct localizations of FgExosc2, FgExosc3, FgExosc5, FgExosc8, FgExosc9, FgExosc10, FgExosc11, and FgExoscA in F. graminearum but is required for the localization of FgExosc4, FgExosc6, and FgExosc7.

## DISCUSSION

The RNA exosome complex is a conserved, multisubunit RNase complex that contributes to the 3′-to-5′ processing and degradation of RNAs in mammalian cells (1). However, the biological functions of the RNA exosome complex have not been reported in plant-pathogenic fungi, and how this complex regulates the fungal development and pathogenicity remain unclear. We identified 12 components of RNA exosome complex in the wheat fungal pathogen F. graminearum. We first found that all 12 subunits are all localized in the nucleus and that they form a complex through interactions. *FgEXOSC1* and *FgEXOSCA* were successfully knocked out, while we could not obtain mutants of the other 10 genes after several attempts. FgExosc1 and FgExoscA were both found to be required for the vegetative growth, conidiation, DON production, and pathogenicity of F. graminearum. Furthermore, *FgEXOSC1* is involved in ncRNA processing, rRNA metabolism and processing, and ribosome and ribonucleoprotein complex biogenesis.

In eukaryotes, the RNA exosome complex consists of about 11 components (one may have multiple subunits) (1, 2, 5, 56). So far, the components of the RNA exosome complex in fungi have not been well investigated. In this study, 12 candidate components of RNA exosome complex were identified; of these, FgExoscA is a specific component that is widely distributed in fungi, but its homologs are absent in plant and mammalian cells. These 12 candidate components have similar localizations. Furthermore, subcellular localization, GFP pulldown, and co-IP experiments all showed that FgExosc1 associates with the other RNA exosome components. Taking these observations together, we speculated that these 12 candidate components form the RNA exosome complex in F. graminearum.

In yeast, humans, and fruit flies, RNA exosome complex is a key factor in growth and development (23, 57, 58). Deletion of *AtCSL4* (*EXOSC1*) does not cause significant phenotypic changes, but homozygous mutations of the remaining components are deadly, while heterozygous mutations lead to different phenotypic defects (10, 26, 27, 59, 60). Deletion of *ScRRP6* (*EXOSC10*) in yeast leads to slow growth of the mutants, while the other components (ScCsl4, ScRrp4, ScRrp40, ScRrp41, ScRrp42, ScRrp43, ScRrp44, ScRrp45, ScRrp46, and ScMtr3) are all essential for the survival of the organism (30, 32, 61). Unlike in yeast, FgExosc10, the homolog protein of ScRrp6, is essential in F. graminearum. In contrast, FgExosc1, the homolog protein of yeast ScCsl4, is dispensable in F. graminearum. Collectively, these results indicate that different RNA exosome components have different functions in the growth and development of different organisms. Through real-time PCR, we found that deletions of *FgEXOSC1* and *FgEXOSCA* resulted in decreased expression of some of the other components of RNA exosome. Furthermore, disruption of *FgEXOSC1* affected the localizations of FgExosc4, FgExosc6, and FgExosc7. These results suggest that *FgEXOSC1* and *FgEXOSCA* deletions may affect the stability of the RNA exosome complex.

There are different forms of RNA exosome in the nucleus and cytoplasm that perform RNA processing or degradation functions in yeast (4). However, our result showed that all the components of RNA exosome complex in F. graminearum are mainly localized in the nucleus, although FgExosc1, FgExosc2, FgExosc3, FgExosc4, FgExosc5, FgExosc6, FgExosc7, FgExosc8, and FgExosc9 also have weak fluorescence signals in the cytoplasm, while the other three subunits (FgExosc10, FgExosc11, and FgExoscA) could not be detected in the cytoplasm. In yeast, Dis3/Rrp44 (Exosc11) is localized in both the nucleus and cytoplasm, while Rrp6 (Exosc10) is present only in the nucleus; in humans, Rrp6 is similarly present in both the nucleus and cytoplasm (62), suggesting different localization pattern of the RNA exosome in F. graminearum.

The RNA exosome complex not only regulates the processing of rRNAs, tRNAs, snRNAs, and snoRNAs but also degrades mRNA and misprocessed RNA, playing an important role in RNA surveillance (63). The RNA exosome has been shown to be the primary regulatory element after transcription, which controls cell differentiation by regulating the transcriptome and proteome (64). In humans and yeast, the RNA exosome complex processes all nascent RNA in the nucleus, especially pre-rRNA, and mutations in any of the exosome complex subunits lead to accumulation of pre-rRNAs within the cell (28). Cytoplasmic RNA exosome complex participates in the quality control and degradation of mRNA, maintaining the stability of mRNA *in vivo*. Eukaryotic rRNAs are transcribed, folded, modified, and processed to form ribosomes through a complex series of assembly and maturation pathways under the mediation of ribosomal assembly factors (AFs) and snoRNAs, bound to approximately 80 ribosome proteins (65, 66). In the present study, RNA-seq analysis of the transcriptome of the wild type and Δ*Fgexosc1* mutant under nutritional conditions revealed that the deletion of *FgEXOSC1* caused the expression of 1,651 genes to be upregulated, while 1,788 genes were downregulated. These differential levels of gene expression are mainly linked to ncRNA processing, rRNA metabolism and processing, and ribosome and ribonucleoprotein complex biogenesis, indicating that the RNA exosome complex of F. graminearum plays an important role in ribosome biogenesis as well as processing of rRNA and ncRNA. In addition, we found that some secreted pathogenesis-related genes were downregulated (Fig. S6), including *OSP24*, *OSP25*, and *OSP44* (67). Among those genes, *OSP24* was identified as an important virulence factor of F. graminearum which modulates host immunity by mediating proteasomal degradation of TaSnRK1α (67), suggesting that the RNA exosome may be involved in regulating the functions of virulence factors at transcription level in F. graminearum.

In summary, our study identified 12 components of the RNA exosome complex in F. graminearum. All the RNA exosome components are localized in the nucleus. FgExosc1 and FgExoscA are both required for vegetative growth, sexual reproduction, DON production, and pathogenicity. FgExosc1 is involved in ncRNA processing, rRNA and ncRNA metabolism, ribosome biogenesis, and ribonucleoprotein complex biogenesis. FgExosc1 associates with the other components of RNA exosome complex to form the RNA exosome complex in F. graminearum. Our findings provide new insights into the roles of RNA exosome in regulating fungal development and pathogenicity.

## MATERIALS AND METHODS

### Fungal strains and culture conditions.

Fusarium graminearum PH-1 was used as the wild type, from which all the mutants were generated (Table S3). All the strains were cultured on CM, starch yeast medium (SYM), and minimal medium (MM) at 28°C for 3 days (68). For cultivation in liquid medium, mycelia from 2-day-old colonies were transferred into liquid CM and incubated for 2 days with shaking (180 rpm) at 28°C.

### Gene deletion, GFP/mCherry fusion vector constructions, and complementation.

Protoplast preparation and fungal transformation of F. graminearum were performed by following standard protocols (69). *FgEXOSC1* (FGSG_13120) and *FgEXOSCA* (FGSG_08866) genes were deleted using split-marker approach (70). The primers used to amplify the flanking sequences for each gene are listed in Table S4. Knockout candidates were verified by Southern blotting. For complementation, the resulting Δ*Fgexosc1* and Δ*FgexoscA* mutants were complemented with *GFP-FgEXOSC1* and *FgEXOSCA-GFP* vectors, respectively. In brief, the *GFP-FgEXOSC1* vector was generated by amplifying the open reading frame (ORF) and native promoter of *FgEXOSC1* and *GFP* genes using the primer pair in Table S4. The amplicons were ligated and then cloned into a pKNT vector and the product was sequenced for verification. The same method was used to construct the *mCherry-FgEXOSC1* vector. The *FgEXOSCA-GFP* vector was generated by amplifying the ORF and native promoter of *FgEXOSCA* using the primer pair in Table S4. The gene sequence was tagged with GFP at its C terminus and then cloned into a pKNTG2 vector and finally sequenced for verification. The same method was used to generate the GFP fusion constructs for other RNA exosome complex subunits.

### Assays for asexual reproduction, sexual reproduction, and ascospore discharge.

To assay asexual reproduction, PH-1 and the various mutants were inoculated into liquid carboxymethyl cellulose (CMC) medium to induce conidiation (9). The number of conidia produced by each strain was determined 3 days after incubation at 28°C using a hemacytometer (Qiujing, Shanghai, China) under an Olympus BX53F microscope (Olympus, Tokyo, Japan). To induce sexual reproduction, the wild type and the mutants were inoculated on carrot agar medium and incubated at 28°C for 5 to 7 days, and 1 mL of 2.5% sterile Tween 60 was pressed gently into each plate (Macklin, Shanghai, China). All of the sexual reproduction-induced cultures were incubated at 22°C under black light (F20T8/BLB [Danqi, Shanghai, China]; wavelength, 365 nm). The perithecia formed were photographed and recorded after 10 days. As for the ascospore discharge, the 7-day-old perithecia were extracted from the plates using a 0.5-cm hole punch, placed on hydrophobic slides, put in a black box to moisturize, and incubated at 22°C under black light for 3 days. Each experiment was independently repeated three times.

### Plant infection and DON production assays.

Infection assays on flowering wheat heads and wheat coleoptiles were conducted as previously described (68, 71). Mycelial agar blocks (5 mm in diameter) were inoculated into liquid trichothecene biosynthesis induction (TBI) medium for 7 days in the dark to assay DON production. The DON produced was quantified using a vomitoxin detection kit (enzyme-linked immunosorbent assay) and a Berthold multifunctional microplate reader.

### qRT-PCR.

For quantitative real-time PCR (qRT-PCR) of DON biosynthesis genes, PH-1 and the Δ*Fgexosc1* mutant were inoculated in liquid TBI for 3 days. Total RNA was isolated from mycelia using an Eastep total RNA extraction kit, and first-strand cDNA was synthesized using Moloney murine leukemia virus (M-MLV) reverse transcriptase as previously reported (72). For relative expression, the data were analyzed using the 2^−ΔΔ^*^CT^* (cycle threshold) method (73). The F. graminearum housekeeping genes for actin (FGSG_07335) and β-tubulin (FGSG_06611) were used as the endogenous reference genes. A similar method was used to analyze the relative expression levels of *FgEXOSC2*, *FgEXOSC3*, *FgEXOSC4*, *FgEXOSC5*, *FgEXOSC6*, *FgEXOSC7*, *FgEXOSC8*, *FgEXOSC9*, *FgEXOSC10*, and *FgEXOSC11* in Δ*Fgexosc1* and Δ*FgexoscA* mutants. All experiments and qRT-PCR assays were repeated three times.

### RNA-seq analysis.

Mycelia from PH-1 and the Δ*Fgexosc1* mutant were cultured in CM for 2 and 4 days, respectively. Mycelia from the resulting cultures were then harvested and stored at −80°C for future use. RNA was extracted and detected using the RNA Nano 6000 assay kit of the Bioanalyzer 2100 system (Agilent Technologies, California, USA). The RNA library was sequenced on an Illumina NovaSeq platform and 150-bp paired-end reads were generated. Reference genome and gene model annotation files were downloaded directly from genome website. The mapped reads of each sample were assembled by StringTie (v1.3.3b) (74) in a reference-based approach. Differential expression analysis of two conditions/groups was performed using DESeq2 R package (1.20.0). Gene Ontology (GO) enrichment analysis of the differentially expressed genes was conducted using cluster Profiler R package, and the same package was used to test the statistical enrichment of the differentially expressed genes in KEGG pathways. These transcriptome analyses were completed by Novogene Bioinformatics Institute (Beijing, China).

### Affinity capture-mass spectrometry analysis, immunoblotting, and co-IP assays.

Briefly, GFP-FgExosc1 and PH-1-GFP strains were cultured in liquid CM for 2 days with shaking (180 rpm) at 28°C. Mycelia were then harvested and stored at −80°C. An affinity purification assay was then carried out as previously reported (75). For immunoblot analysis, total proteins were isolated from vegetative hyphae as described above. The extracted proteins were separated on 10% SDS-PAGE gels and then transferred onto a polyvinylidene difluoride (PVDF) membrane with a Bio-Rad electroblotting apparatus. The proteins were finally detected by anti-GFP (M20004L) and anti-mCherry (NBP1-96752) as previously reported (75).

### BiFC assays.

All the primers used for BiFC assays are listed in Table S4. *FgEXOSC1* and *FgEXOSCA* were cloned into pCX62-cYFP vector (at the C-terminal portion of the yellow fluorescent protein [YFP]). *FgEXOSC1*, -*2*, -*3*, -*4*, -*5*, -*6*, -*7*, -*8*, -*9*, -*10*, -*11*, and -*A* were cloned into the pKNT-nYFP vector (at the N-terminal portion of the YFP fluorescent protein). The resulting constructs were verified by sequencing and then cotransformed into the protoplasts of PH-1 in pairs (*FgEXOSC1-cYFP* vector cotransformed with each of the vectors *FgEXOSC2-nYFP*, -*3-nYFP*, -*4-nYFP*, -*5-nYFP*, -*6-nYFP*, -*7-nYFP*, -*8-nYFP*, -*9-nYFP*, -*10-nYFP*, -*11-nYFP*, and -*A-nYFP* or the *FgEXOSCA-cYFP* vector cotransformed with each of the vectors *FgEXOSC2-nYFP*, -*3-nYFP*, -*4-nYFP*, -*5-nYFP*, -*6-nYFP*, -*7-nYFP*, -*8-nYFP*, -*9-nYFP*, -*10-nYFP*, and -*11-nYFP*). YFP signals were observed using a Nikon A1R laser scanning confocal microscope. GFP and YFP excitations were detected at 488-nm light (emission, 525/40 nm).

### Data availability.

The RNA-seq data were deposited in the NCBI BioProject database under accession number PRJNA894853.
