# Supplementary material for: Genome-Wide Characterization of the RNA Exosome Complex in Relation to Growth, Development, and Pathogenicity of Fusarium graminearum
Source: Microbiol Spectr. 2023 May 9;11(3):e05058-22. doi: 10.1128/spectrum.05058-22 (PMC10269758; doi:10.1128/spectrum.05058-22)
Supplement: Supplemental file 1 — Supplemental material. Download spectrum.05058-22-s0001.pdf, PDF file, 1.1 MB [file spectrum.05058-22-s0001.pdf]

SUPPLEMENTAL MATERIAL

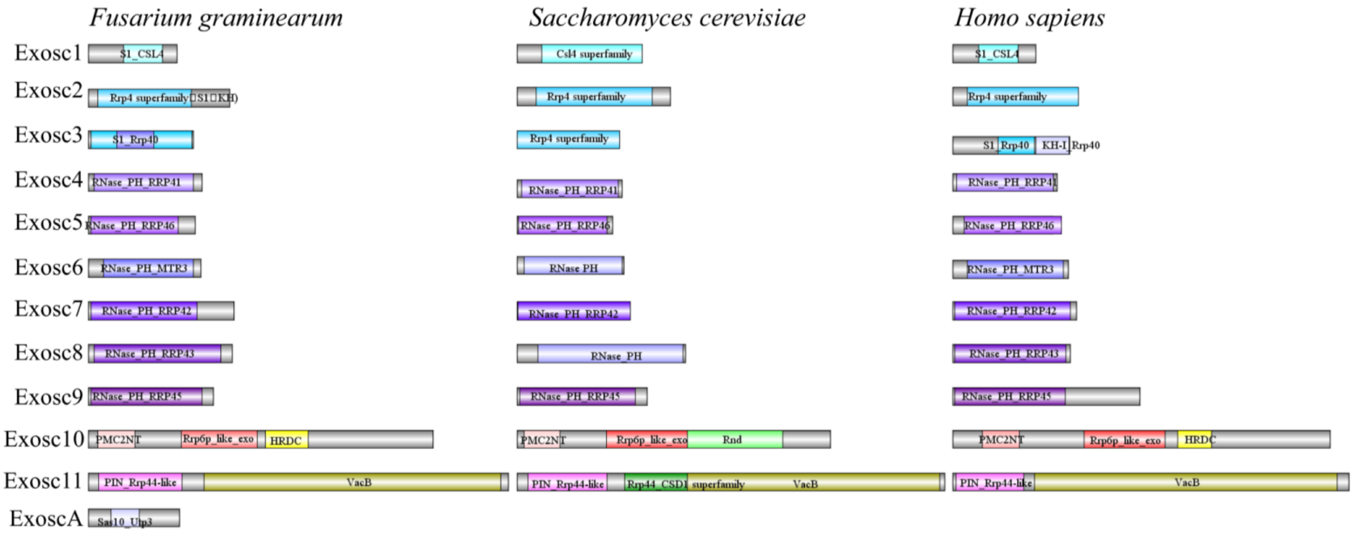

FIG S1 Analysis of the conserved domains of RNA exosome complex in *F. graminearum*, *Saccharomyces cerevisiae* and *Homo sapiens.*

The conservative domains present in the various subunits of RNA exosome complex in *F. graminearum*, *Saccharomyces cerevisiae* and *Homo sapiens* were predicted.

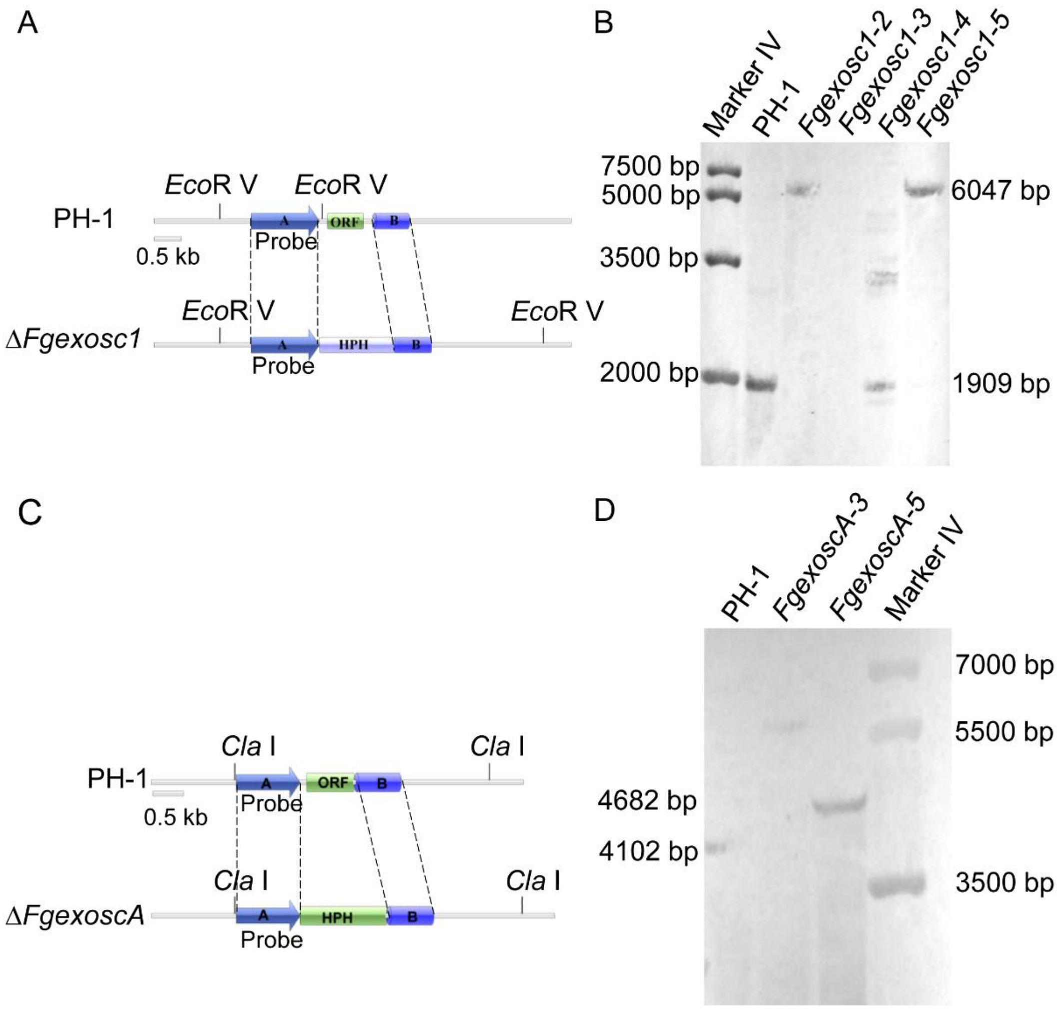

FIG S2 Southern blot confirmation of *FgEXOSC1* and *FgEXOSCA* deletions in *F. graminearum.*

(A) The split-marker approach was used to delete the *FgEXOSC1* gene in *F. graminearum*. (B) For targeted gene deletion of *FgEXOSC1*, *Eco*R V was used to digest the genomic DNAs, which finally showed a 1909 bp band in the wild type and a 6047 bp band in the mutants. (C) The split-marker approach was used to delete *FgEXOSCA*. (D) For targeted gene deletion of *FgEXOSCA*, *Cla* I was used to digest the genomic DNAs, which showed a 4102 bp band in the wild type and a 4682 bp band in the mutants.

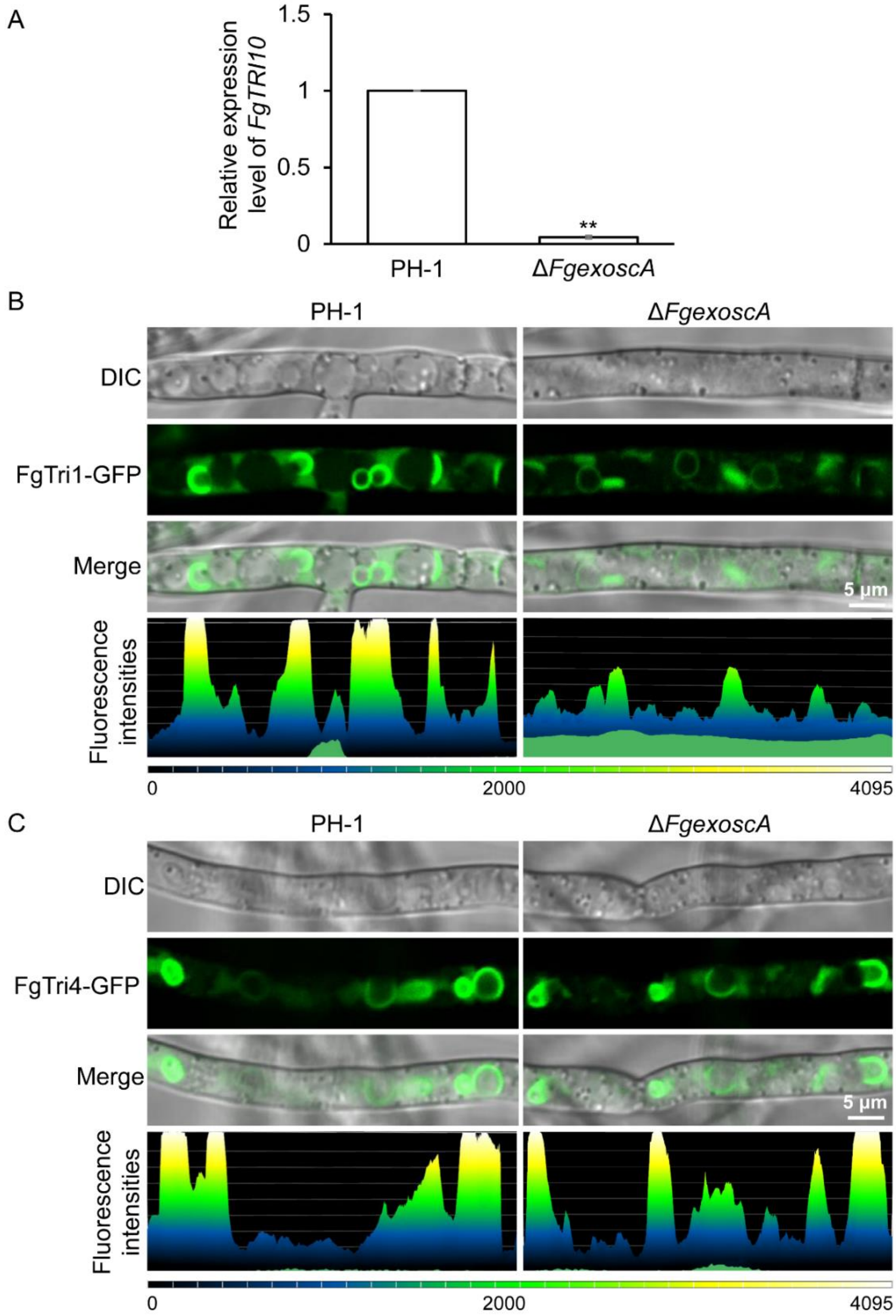

FIG S3 Role of FgExoscA in DON production of *F. graminearum*.

(A) The relative expression levels of the DON biosynthesis gene *FgTRI10* in PH-1 and the Δ*FgexoscA* mutant (***P*< 0.01). (B-C) The localizations of FgTri1-GFP and FgTri4-GFP in PH-1 and the Δ*FgexoscA* strains. Fluorescence intensities of FgTri1-GFP and FgTri4-GFP in PH-1 and the Δ*FgexoscA* mutant are shown.

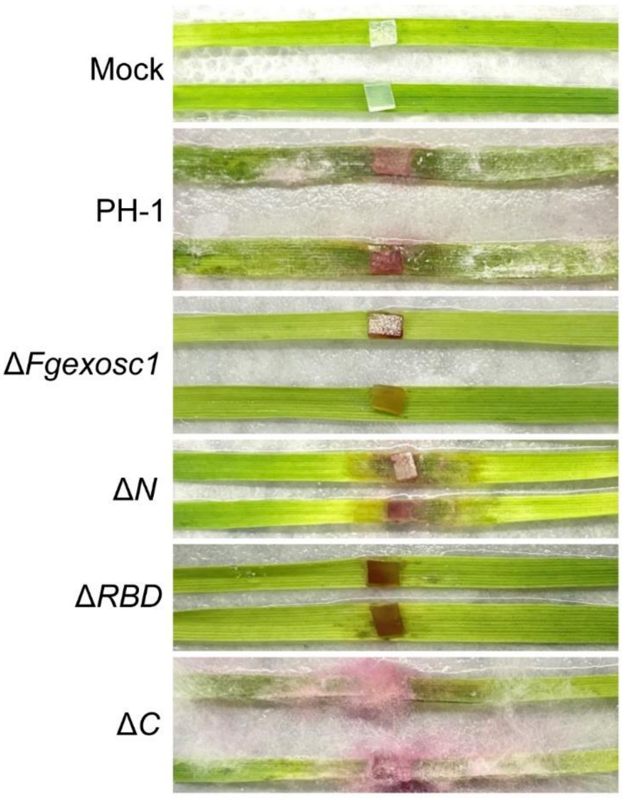

FIG S4 Analyses of the pathogenicity of *FgEXOSC1* domain deletion mutants on wheat seedling leaves.

PH-1, the Δ*Fgexosc1*, Δ*N*, Δ*RBD* and Δ*C* strains were inoculated on wheat seedling leaves for 5 days. The RBD domain and N-terminal region of FgExosc1 are both required for pathogenicity of *F. graminearum* to wheat seedling leaves.

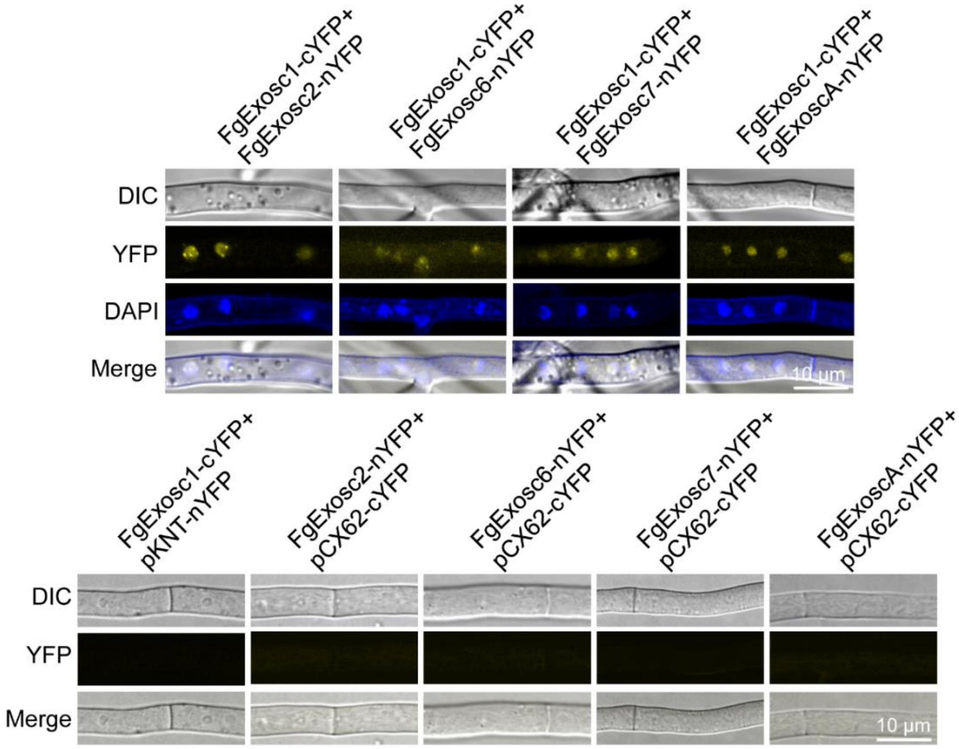

FIG S5 Bimolecular fluorescence complementation (BiFC) assay showing interaction relationship of FgExosc1 with other components of RNA exosome.

FgExosc1 directly interacts with FgExosc2, FgExosc6, FgExosc7, and FgExoscA as evidenced by BiFC experiments.

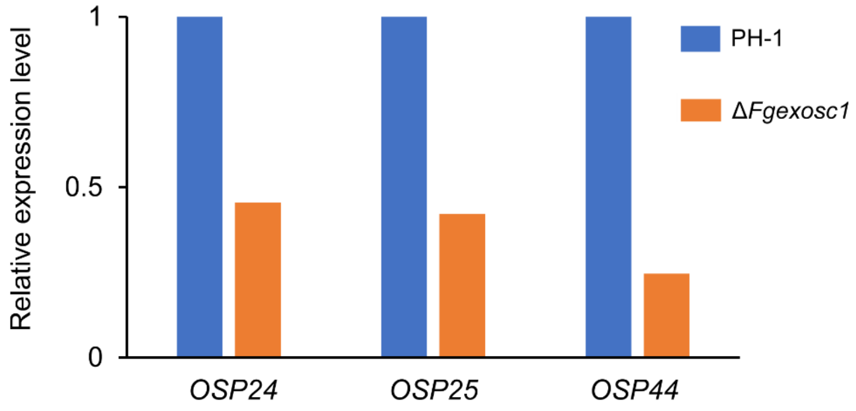

FIG S6 The relative expression levels of *OSP24*, *OSP25* and *OSP44* in the wild-type strain PH-1 and the Δ*Fgexosc1* mutant.

TABLE S1 Identification of the subunits of exosome complex in *Fusarium graminearum*

Gene ID in *Fusarium graminearum*	Name	Homologous gene in *Saccharomyces cerevisiae*	Per.Ident	Homologous gene in *Homo sapiens*	Per.Ident
FGSG_13120	FgExosc1	NP_014167.1 ScCsl4	31.74%	EAW49936.1 HoExosc1	43.94%
FGSG_09961	FgExosc2	NP_011936.1 ScRrp4	44.33%	AAH00747.1 HoExosc2	39.38%
FGSG_08645	FgExosc3	NP_014499.2 ScRrp40	34.15%	EAW58264.1 HoExosc3	30.37%
FGSG_09363	FgExosc4	NP_011711.3 ScRrp41	37.55%	AAH02777.1 HoExosc4	36.21%
FGSG_01091	FgExosc5	NP_011609.2 ScRrp46	35.75%	EAW57038.1 HoExosc5	25.13%
FGSG_04277	FgExosc6	NP_011674.3 ScMtr3	28.37%	-	
-		-		AAH52252.1 HoExosc6	29.96%
FGSG_10879	FgExosc7	NP_010172.1 ScRrp42	32.92%	-	
-		-		NP_055819.2 HoExosc7	28.29%
FGSG_08578	FgExosc8	-		NP_852480.1 HoExosc8	33.09%
-		NP_009964.2 ScRrp43	24.83%	-	
FGSG_05509	FgExosc9	NP_010566.1 ScRrp45	43.93%	AAI42979.1 HoExosc9	43.57%
FGSG_06049	FgExosc10	NP_014643.1 ScRrp6	38.18%	EAW71678.1 HoExosc10	34.37%

FGSG_01184	FgExosc11	NP_014621.1<br>ScDis3	53.61%	NP_001121698.1<br>HoExosc11	43.42%
FGSG_08866	FgExoscA	-		-	

-, ‘no homologous protein’.

TABLE S2 Accession numbers of all the exosome components

Protein names	Accession Numbers	Species
AtCsl4	BAC42612.1	*Arabidopsis thaliana*
AnCsl4	XP_001401124.2	*Aspergillus niger*
MoExosc1	XP_003711058.1	*Pyricularia oryzae 70-15*
ScCsl4	KAG2512570.1	*Saccharomyces cerevisiae*
HoExosc1	EAW49936.1	*Homo sapiens*
FgExosc1	XP_011327073.1	*Fusarium graminearum PH-1*
AtRrp4	Q9ZVT7.1	*Arabidopsis thaliana*
AnExosc2	GAQ44761.1	*Aspergillus niger*
MoExosc2	XP_003715055.1	*Pyricularia oryzae 70-15*
ScRrp4	NP_011936.1	*Saccharomyces cerevisiae*
HoExosc2	AAH00747.1	*Homo sapiens*
FgExosc2	XP_011318870.1	*Fusarium graminearum PH-1*
AtRrp40a	NP_565592.	*Arabidopsis thaliana*
AnExosc3	GAQ39206.1	*Aspergillus niger*
MoExosc3	SEHA46151.1	*Pyricularia oryzae 70-15*
ScRrp40	NP_014499.2	*Saccharomyces cerevisiae*
HoExosc3	EAW58264.1	*Homo sapiens*
FgExosc3	XP_011320097.1	*Fusarium graminearum PH-1*
AtRRP41	AAF04590.1	*Arabidopsis thaliana*
AnExosc4	KAI2831469.1	*Aspergillus niger*
MoExosc4	XP_003709504.1	*Pyricularia oryzae 70-15*
ScSki6	NP_011711.3	*Saccharomyces cerevisiae*
HoExosc4	AAH02777.1	*Homo sapiens*
FgExosc4	XP_011328382.1	*Fusarium graminearum PH-1*
AtRrp46	Q9LX74.1	*Arabidopsis thaliana*
AnExosc5	XP_001390391.1	*Aspergillus niger*
MoExosc5	XP_003710038.1	*Pyricularia oryzae 70-15*
ScRrp46	NP_011609.2	*Saccharomyces cerevisiae*
HoExosc5	AAI07697.1	*Homo sapiens*
FgExosc5	XP_011316853.1	*Fusarium graminearum*

		PH-1
AtMtr3	NP_001320075.1	*Arabidopsis thaliana*
AnExosc6	XP_001397160.1	*Aspergillus niger*
MoExosc6	XP_003715270.1	*Pyricularia oryzae 70-15*
ScMtr3	NP_011674.3	*Saccharomyces cerevisiae*
HoExosc6	EAW51841.1	*Homo sapiens*
FgExosc6	XP_011321341.1	*Fusarium graminearum PH-1*
AtRrp42	NP_566318.1	*Arabidopsis thaliana*
AnExosc7	XP_001399338.1	*Aspergillus niger*
MoExosc7	XP_003709390.1	*Pyricularia oryzae 70-15*
ScRrp42	NP_010172.1	*Saccharomyces cerevisiae*
HoExosc7	NP_055819.2	*Homo sapiens*
FgExosc7	SCB64963.1	*Fusarium graminearum PH-1*
AtRrp43	NP_176216.1	*Arabidopsis thaliana*
AnExosc8	GAQ45644.1	*Aspergillus niger*
MoExosc8	EHA46356.1	*Pyricularia oryzae 70-15*
ScRrp43	NP_009964.2	*Saccharomyces cerevisiae*
HoExosc8	AAH20773.1	*Homo sapiens*
FgExosc8	XP_011320172.1	*Fusarium graminearum PH-1*
AtRrp45a	Q9LDM2.1	*Arabidopsis thaliana*
AnExosc9	XP_001395525.1	*Aspergillus niger*
MoExosc9	XP_003713883.1	*Pyricularia oryzae 70-15*
ScRrp45	NP_010566.1	*Saccharomyces cerevisiae*
HoExosc9	AAI42979.1	*Homo sapiens*
FgExosc9	XP_011324056.1	*Fusarium graminearum PH-1*
AtRrp6L1	ABX52079.1	*Arabidopsis thaliana*
AnExosc10	GAQ43193.1	*Aspergillus niger*
MoExosc10	XP_003718068.1	*Pyricularia oryzae 70-15*
ScRrp6	NP_014643.1	*Saccharomyces cerevisiae*
HoExosc10	AAH39901.1	*Homo sapiens*
FgExosc10	XP_011324669.1	*Fusarium graminearum PH-1*
AtRrp44a	Q9SHL7.2	*Arabidopsis thaliana*

AnExosc11	GAQ34026.1	*Aspergillus niger*
MoExosc11	XP_003718924.1	*Pyricularia oryzae 70-15*
ScDis3	NP_014621.1	*Saccharomyces cerevisiae*
HoExosc11	NP_055768.3	*Homo sapiens*
FgExosc11	XP_011316956.1	*Fusarium graminearum PH-1*
FgExoscA	CEF77318.1	*Fusarium graminearum PH-1*
AnExoscA	XP_001394954.2	*Aspergillus niger*
MoExoscA	XP_003721020.1	*Pyricularia oryzae 70-15*
FoExoscA	EWZ39269.1	*Fusarium oxysporum*
SsExoscA	XP_001586071.1	*Sclerotinia sclerotiorum*
UmExoscA	XP_011386404.1	*Ustilago maydis*
AnExoscA	XP_001394954.2	*Aspergillus niger*
VdExoscA	XP_009651425.1	*Verticillium dahliae*

TABLE S3 Strains used in this study

Strain	Description	References
PH-1	Wild type	[1]
Δ*Fgexosc1*	FgExosc1 deletion mutant of PH-1	This study
Δ*FgexoscA*	FgExoscA deletion mutant of PH-1	This study
Δ*Fgexosc1*-C	GFP-FgExosc1 transformant of Δ*Fgexosc1* mutant	This study
Δ*FgexoscA*-C	FgExoscA-GFP transformant of Δ*FgexoscA* mutant	This study
PH-1: mCherry-FgExosc1	mCherry-FgExosc1 transformant of PH-1	This study
PH-1: FgExosc1-GFP	FgExosc1-GFP transformant of PH-1	This study
PH-1: FgExosc2-GFP	FgExosc2-GFP transformant of PH-1	This study
PH-1: FgExosc3-GFP	FgExosc3-GFP transformant of PH-1	This study
PH-1: FgExosc4-GFP	FgExosc4-GFP transformant of PH-1	This study
PH-1: FgExosc5-GFP	FgExosc5-GFP transformant of PH-1	This study
PH-1: FgExosc6-GFP	FgExosc6-GFP transformant of PH-1	This study
PH-1: FgExosc7-GFP	FgExosc7-GFP transformant of PH-1	This study
PH-1: GFP-FgExosc8	GFP-FgExosc8 transformant of PH-1	This study
PH-1: FgExosc9-GFP	FgExosc9-GFP transformant of PH-1	This study
PH-1: GFP-FgExosc10	GFP-FgExosc10 transformant of PH-1	This study
PH-1: FgExosc11-GFP	FgExosc11-GFP transformant of PH-1	This study
Δ*Fgexosc1*: FgExosc2-GFP	FgExosc2-GFP transformant of Δ*Fgexosc1*	This study
Δ*Fgexosc1*: FgExosc3-GFP	FgExosc3-GFP transformant of Δ*Fgexosc1*	This study
Δ*Fgexosc1*: FgExosc4-GFP	FgExosc4-GFP transformant ofΔ*Fgexosc1*	This study
Δ*Fgexosc1*: FgExosc5-GFP	FgExosc5-GFP transformant of Δ*Fgexosc1*	This study
Δ*Fgexosc1*: FgExosc6-GFP	FgExosc6-GFP transformant of Δ*Fgexosc1*	This study
Δ*Fgexosc1*: FgExosc7-GFP	FgExosc7-GFP transformant of Δ*Fgexosc1*	This study
Δ*Fgexosc1*: GFP-FgExosc8	GFP-FgExosc8 transformant of Δ*Fgexosc1*	This study
Δ*Fgexosc1*: FgExosc9-GFP	FgExosc9-GFP transformant of Δ*Fgexosc1*	This study
Δ*Fgexosc1*: GFP-FgExosc10	GFP-FgExosc10 transformant of Δ*Fgexosc1*	This study
Δ*Fgexosc1*: FgExosc11-GFP	FgExosc11-GFP transformant of Δ*Fgexosc1*	This study

Δ*Fgexosc1*: FgExoscA-GFP	FgExoscA-GFP transformant of Δ*Fgexosc1*	This study
PH-1: FgTri1-GFP	FgTri1-GFP transformant of PH-1	[2]
Δ*Fgexosc1*: FgTri1-GFP	FgTri1-GFP transformant of Δ*Fgexosc1*	This study
Δ*FgexoscA*: FgTri1-GFP	FgTri1-GFP transformant of Δ*FgexoscA*	This study
PH-1: FgTri4-GFP	FgTri4-GFP transformant of PH-1	[2]
Δ*Fgexosc1*: FgTri4-GFP	FgTri4-GFP transformant of Δ*Fgexosc1*	This study
Δ*FgexoscA*: FgTri4-GFP	FgTri4-GFP transformant of Δ*FgexoscA*	This study
GFP-FgExosc1+FgNucleolin-mCherry	GFP-FgExosc1 and FgNucleolin-mCherry transformant of PH-1	This study
GFP-FgExosc1+FgNcbp2-mCherry	GP-FgExosc1 and FgNcbp2-mCherry transformant of PH-1	This study
mCherryFgExosc1+FgExosc2-GFP	mCherry-FgExosc1 and FgExosc2-GFP transformant of PH-1	This study
mCherry-FgExosc1+FgExosc3-GFP	mCherry-FgExosc1 and FgExosc3-GFP transformant of PH-1	This study
mCherry-FgExosc1+FgExosc4-GFP	mCherry-FgExosc1 and FgExosc4-GFP transformant of PH-1	This study
mCherry-FgExosc1+FgExosc5-GFP	mCherry-FgExosc1 and FgExosc5-GFP transformant of PH-1	This study
mCherry-FgExosc1+FgExosc6-GFP	mCherry-FgExosc1 and FgExosc6-GFP transformant of PH-1	This study
mCherry-FgExosc1+FgExosc7-GFP	mCherry-FgExosc1 and FgExosc7-GFP transformant of PH-1	This study
mCherry-FgExosc1+GFP-FgExosc8	mCherry-FgExosc1 and GFP-FgExosc8 transformant of PH-1	This study
mCherry-FgExosc1+FgExosc9-GFP	mCherry-FgExosc1 and FgExosc9-GFP transformant of PH-1	This study
mCherry-FgExosc1+GFP-FgE	mCherry-FgExosc1 and GFP-FgExosc10	This study

xosc10	transformant of PH-1	
mCherry-FgExosc1+FgExosc11-GFP	mCherry-FgExosc1 and FgExosc11-GFP transformant of PH-1	This study
mCherry-FgExosc1+FgExoscA-GFP	mCherry-FgExosc1 and FgExoscA-GFP transformant of PH-1	This study
FgExosc1-cYFP+FgExosc2-nYFP	FgExosc1-cYFP and FgExosc2-nYFP transformant of PH-1	This study
FgExosc1-cYFP+pKNT-nYFP	FgExosc1-cYFP and pKNT-nYFP transformant of PH-1	This study
pCX62-cYFP+FgExosc2-nYFP	pCX62-cYFP and FgExosc2-nYFP transformant of PH-1	This study
FgExosc1-cYFP+FgExosc6-nYFP	FgExosc1-cYFP and FgExosc6-nYFP transformant of PH-1	This study
pCX62-cYFP+FgExosc6-nYFP	pCX62-cYFP and FgExosc6-nYFP transformant of PH-1	This study
FgExosc1-cYFP+FgExosc7-nYFP	FgExosc1-cYFP and FgExosc7-nYFP transformant of PH-1	This study
pCX62-cYFP+FgExosc7-nYFP	pCX62-cYFP and FgExosc7-nYFP transformant of PH-1	This study
FgExosc1-cYFP+FgExoscA-nYFP	FgExosc1-cYFP and FgExoscA-nYFP transformant of PH-1	This study
pCX62-cYFP+FgExoscA-nYFP	pCX62-cYFP and FgExoscA-nYFP transformant of PH-1	This study
FgExoscA-cYFP+pKNT-nYFP	FgExoscA-cYFP and pKNT-nYFP transformant of PH-1	This study
FgExoscA-cYFP+FgExosc2-nYFP	FgExoscA-cYFP and FgExosc2-nYFP transformant of PH-1	This study
FgExoscA-cYFP+FgExosc3-nYFP	FgExoscA-cYFP and FgExosc3-nYFP	This study

	transformant of PH-1	
FgExoscA-cYFP+FgExosc8-nYFP	FgExoscA-cYFP and FgExosc8-nYFP transformant of PH-1	This study
pCX62-cYFP+pKNT-nYFP	pCX62-cYFP and pKNT-nYFP transformant of PH-1	This study
pCX62-cYFP+FgExosc2-nYFP	pCX62-cYFP and FgExosc2-nYFP transformant of PH-1	This study
pCX62-cYFP+FgExosc3-nYFP	pCX62-cYFP and FgExosc3-nYFP transformant of PH-1	This study
pCX62-cYFP+FgExosc8-nYFP	pCX62-cYFP and FgExosc8-nYFP transformant of PH-1	This study

1. Cuomo CA, Gueldener U, Xu JR, Trail F, Turgeon BG, et al. (2007) The Fusarium graminearum genome reveals a link between localized polymorphism and pathogen specialization. Science 317: 1400-1402.
2. Yang C, Li J, Chen X, Zhang X, Liao D, et al. (2020) FgVps9, a Rab5 GEF, Is Critical for DON Biosynthesis and Pathogenicity in. Frontiers In Microbiology 11: 1714.

TABLE S4 The primers used in this study

Primers	Sequence ((5'→3')	Application
FgExosc1AF	GCAGGATCGGGATTGGTT	*FgEXOSC1* deletion and southern probe
FgExosc1AR	TTGACCTCCACTAGCTCCAGCCAAGCCGTCGTTCGGGCTGTTCTA	
FgExosc1BF	GAATAGAGTAGATGCCGACCGCGGGTTTACAGCCACAAAGTTCTAATC	
FgExosc1BR	CGGTCCAAGTGTCCCTAT	*FgEXOSC1* deletion
FgExosc1OF	CCGGTTACCAAGTTTGCG	
FgExosc1OR	GGCTCACTGATGCCTGTTT	
FgExosc1UA	TATCTCGTCCCAGTTCACA	
FgExosc2AF	AGGTGGACTGACAAGGGAC	
FgExosc2AR	TTGACCTCCACTAGCTCCAGCCAAGCCAGATGAAGATGGCGAGGC	
FgExosc2BF	GAATAGAGTAGATGCCGACCGCGGGTTTGGCTAACTACAAACGACA	*FgEXOSC2* deletion
FgExosc2BR	GCGTACCTTCTTCCCAAA	
FgExosc2OF	CTCAGTCCTCAGCCTCCT	
FgExosc2OR	CAACCCATTCTCAACCAG	
FgExosc2UA	CGATGTCGCAATGAATAAGAGG	
FgExosc3AF	GGGACGGGCATCATAAGC	
FgExosc3AR	TTGACCTCCACTAGCTCCAGCCAAGCCTGAGCAGAAGCGGCGTAG	
FgExosc3BF	GAATAGAGTAGATGCCGACCGCGGGTTACACGTCTAGCTCGTTCTT	*FgEXOSC3* deletion
FgExosc3BR	GCTTGATACACCTTCTCCC	
FgExosc3OF	TCGCATCCTAAGAAACCT	
FgExosc3 OR	CCCTGCCTACAGCAATAA	
FgExosc3UA	GGTGGTGACAGCAGGGTGA	
FgExosc4AF	TAACCCATCTTGTAGGCG	
FgExosc4AR	TTGACCTCCACTAGCTCCAGCCAAGCCTTGGAACATTTGCTGCTT	*FgEXOSC4* deletion
FgExosc4BF	GAATAGAGTAGATGCCGACCGCGGGTTGTTCTTTGTTTGATCCGTAG	

FgExosc4BR	CGGACAACCTAACCCTAT	
FgExosc4OF	GACGGAAGAAGATGGAACG	
FgExosc4OR	GACGGTAAGGAATGGAAGT	
FgExosc4UA	GTCGAGTCGAACGGTTAA	
FgExosc5AF	CGATGGGCTTAAAGTCGG	*FgEXOSC5* deletion
FgExosc5AR	TTGACCTCCACTAGCTCCAGCCAAGCCCGCTGTGAA AGGCGGTAT	
FgExosc5BF	GAATAGAGTAGATGCCGACCGCGGGTTTCTTAGCGT AAGCCTTCT	
FgExosc5BR	GCTGTCACCGTCTCAATA	
FgExosc5OF	CTCCCTTTACTGAACCAACA	
FgExosc5OR	TGTCCTTGCCCTCCTCTT	
FgExosc5UA	ATAACATTCGCATCTTACTGG	
FgExosc6AF	AATCTGTATCCGTTAGCC	*FgEXOSC6* deletion
FgExosc6AR	TTGACCTCCACTAGCTCCAGCCAAGCCATTTGCCTT CATTCCTAC	
FgExosc6BF	GAATAGAGTAGATGCCGACCGCGGGTTCCCGACACT CCATCAAAG	
FgExosc6BR	AGGACGCAAATCGCATAA	
FgExosc6OF	GGCGAATGGTATCAGAGC	
FgExosc6OR	TAGCGTCAGACGAAGGTAGG	
FgExosc6UA	GCTGGTTTGGACATACTTG	
FgExosc7AF	GAAGAGGATAGCGACTGG	*FgEXOSC7* deletion
FgExosc7AR	TTGACCTCCACTAGCTCCAGCCAAGCCATGACAATG GCAGCGTAT	
FgExosc7BF	GAATAGAGTAGATGCCGACCGCGGGTTCTTGGGTGT CGGCTTTGC	
FgExosc7BR	CGATTGTGGCTCCGGGTA	
FgExosc7OF	CCTGGCACGAATGGTAGT	
FgExosc7OR	CCCTTCTGAGCCGAGTTA	
FgExosc7UA	GGTTACTGTGGAAGTCGTGGCA	
FgExosc8AF	ATTATTCAGGCGGGTTGT	*FgEXOSC8* deletion
FgExosc8AR	TTGACCTCCACTAGCTCCAGCCAAGCCTGTGG TGGTTGAGATTGTT	

FgExosc8BF	GAATAGAGTAGATGCCGACCGCGGGTTCTAGG GCAGGAGATACCG	
FgExosc8BR	AGAGCCGACAGCGAGAAA	
FgExosc8OF	ACTTTCACCGCATCCATA	
FgExosc8OR	TTGACCTTGCCGTTCTTG	
FgExosc8UA	AGGTTGCTGGTGTCCGAGTT	
FgExosc9AF	TCTTGCCCTTCTCCTCTG	*FgEXOSC9* deletion
FgExosc9AR	TTGACCTCCACTAGCTCCAGCCAAGCCCGGTG AATCGCCACTTAT	
FgExosc9BF	GAATAGAGTAGATGCCGACCGCGGGTTAGTAC ATACGCCAGCCAGAC	
FgExosc9BR	AAGCGATAGGACTTTGAATAGG	
FgExosc9OF	AACCGCTGACGCATTTCT	
FgExosc9OR	ATGAGCCCTCCCTTGTCC	
FgExosc9UA	CGGTGCTGCCAAGGATGA	
FgExosc10AF	AGCTCCTCAAGATTGTTCC	*FgEXOSC10* deletion
FgExosc10AR	TTGACCTCCACTAGCTCCAGCCAAGCCTACGA GACGACAGCGAAT	
FgExosc10BF	GAATAGAGTAGATGCCGACCGCGGGTTTCACT GGTCCCAATTTAC	
FgExosc10OF	CCAACTTCTAACCACCTACC	
FgExosc10OR	CTTGATGTCCGTGTCTGC	
FgExosc10UA	AGAAGAGCGTGGTAAGCG	
FgExosc11AF	ACCTGGTAGTTATGGTTCG	*FgEXOSC11* deletion
FgExosc11AR	TTGACCTCCACTAGCTCCAGCCAAGCCTCGCA AGGCAATAGAAAA	
FgExosc11BF	GAATAGAGTAGATGCCGACCGCGGGTTGCCCA CTCGGTAACAATC	
FgExosc11BR	CGACAAGCGTTTAATCCTA	
FgExosc11OF	ATACTAACGCACTGCTGAAC	
FgExosc11OR	GGAACAAGGACGACAAAA	
FgExosc11UA	TGACATCGCATAAGCCATCC	
FgExoscA-AF	AGCCTCTTGTCCCTCATC	*FgEXOSCA* deletion and southern probe
FgExoscA-AR	TTGACCTCCACTAGCTCCAGCCAAGCT CGCCACAGGTTCTATCT	

FgExoscA-BF	GAATAGAGTAGATGCCGACCGCGGGTT CTAGAGCAACGATATGCCAGTG	*FgEXOSCA* deletion
FgExoscA-BR	AGCAGCGTAAGCGACAGC	
FgExoscA-OF	TTTGCTCGGTAATCTTCA	
FgExoscA-OR	CTTTATCTTTCCCGTCATC	
FgExoscA-UA	GGGTCGCTTGCTTTACAT	
H853	GACAGACGTCGCGGTGAGTT	
YG/F	GATGTAGGAGGGCGTGGATATGTCCT	Gene deletion
HY/R	GTATTGACCGATTCCTTGCGGTCCGAA	
HYG/F	GGCTTGGCTGGAGCTAGTGGAGGTCAA	
HYG/R	AACCCGCGGTCGGCATCTACTCTATTC	
FgExosc1PF	GGGTACCGGGCCCCCCCTCGAGGGTG GGCGTGGCTACATT	GFP-FgExosc1 Complementation and subcellular localization examination
FgExosc1PR	TCCTCGCCCTTGCTCACCATTGCTGTC GGGATTAGATGACGAATT	
FgExosc1GF	CACTCACGGCATGGACGAGCTGTACAA GATGGCCGTCGACGACATCCCC	
FgExosc1GR	CCCCCGGGCTGCAGGAATTCAAGGCA GTATTCTAACAAAGCA	
FgExosc1M-PF	TCACTAAAGGGAACAAAAGCTGGGTGG TGGGCGTGGCTACATT	mCherry-FgExosc1 Complementation and subcellular examination
FgExosc1M-PR	TCCTCGCCCTTGCTCACCATTGCTGTC GGGATTAGATGACGAATT	
FgExosc1M-GF	gcatggacgagctgtacaagATGGCCGTCGAC GACATCCCCTCCG	
FgExosc1M-GR	CCACTAGCTCCAGCGGCGCGCCGAAAA GGCAGTATTCTAACAAAGCA	
FgExosc1-GFPF	agggaacaaaagctgggtaccGGTGGGCGTGG CTACATT	FgExosc1-GFP subcellular localization examination
FgExosc1-GFPR	gcccttgctcaccataagcttGCTAGGCTTTGCG ACTTTTCGAGGC	
FgExosc2 CF	agggaacaaaagctgggtaccCCTTTGCACCTC GAACTT	subcellular localization examination
FgExosc2 CR	GCCGCCGCCGCCGCCAAGCTTGTGAC CAGCCACAGCAGTTGCTAGA	
FgExosc3 CF	agggaacaaaagctgggtaccACCCTTGCCGC CGACTTCA	

FgExosc3 CR	GCCGCCGCCGCCGCCAAGCTTGCTTAGCTCCTTGATGATTTTCCTC	
FgExosc4 CF	agggaacaaaagctgggtaccAAAGACGCCAACATTTCT	
FgExosc4 CR	GCCGCCGCCGCCGCCAAGCTTTCCATCCAAATCCATCACCGCGTCA	
FgExosc5 CF	agggaacaaaagctgggtaccGCCACAAGCATCAAAGCC	
FgExosc5 CR	GCCGCCGCCGCCGCCAAGCTTTTTCCAGTAGAGATCTTCCGCCACT	
FgExosc6 CF	agggaacaaaagctgggtaccGGAGACAAGCGAACACGG	
FgExosc6 CR	GCCGCCGCCGCCGCCAAGCTTGACCGCAAGGATTGATTCGTTGAGA	
FgExosc7 CF	agggaacaaaagctgggtaccGCTGAGCCCAGGGTTCTA	
FgExosc7 CR	GCCGCCGCCGCCGCCAAGCTTTGAGAGCTCGACACCCTCCAAACCA	
FgExosc8 PF	GGGTACCGGGCCCCCCCTCGAGCGTGAGGGAACGCTAAAT	
FgExosc8 PR	TCCTCGCCCTTGCTCACCATTTCTTTTTGAGACGCAGAATTGGAA	
FgExosc8 GF	CACTCACGGCATGGACGAGCTGTACAAGATGGCGTCAACAACAGGACTTACTC	subcellular localization examination
FgExosc8 GR	CCCCCGGGCTGCAGGAATTCCCACTCAGCCCTTTATGC	
FgExosc9 CF	agggaacaaaagctgggtaccTATGGCATCAAGAAAGGTG	
FgExosc9 CR	GCCGCCGCCGCCGCCAAGCTTCCTATCGTTCTCGGCCGTCAACTCT	
FgExosc10 PF	GGGTACCGGGCCCCCCCTCGAGATAAGAAGCAATGGGACG	
FgExosc10 PR	TCCTCGCCCTTGCTCACCATGGTTGCGACAAACAAGTGCGCGTTG	
FgExosc10 GF	CACTCACGGCATGGACGAGCTGTACAAGATGGAGAAGTCCCAGGATTTCAAGT	
FgExosc10 GR	CCCCCGGGCTGCAGGAATTCTTGTAGTCTAAGCCCGTAA	
FgExosc11 CF	agggaacaaaagctgggtaccTCCCAATCGTCCAGTTTA	
FgExosc11 CR	GCCGCCGCCGCCGCCAAGCTTTGCTTCGATCAGCTCCATCTTGACA	
FgExoscA CF	agggaacaaaagctgggtaccTGCTCCGCCGATGTTTCC	
FgExoscA CR	GCCGCCGCCGCCGCCAAGCTTGTTTTTGGACTTGCTTTTCT	

nYFPF	ATGGTGAGCAAGGGCGAGGA	
nYFPR	CATCGTGGCGATGGAGCGGGCCATGATATAGACGTT	BiFC assay
cYFPF	ATGGACAAGCAGAAGAACGGCATC	
cYFPR	GTGGTTCATGACCTTCTGTTTCAGGTCGTTCGGGATCTTGCAGGCCGGGCGCTTGTACAGCTCGTCCAT	
cYFP-FgExosc1-PF	TCACTAAAGGGAACAAAAGCTGGGTGGTGGGCGTGGCTACATT	
cYFP-FgExosc1-PR	GATGCCGTTCTTCTGCTTGTCCATTGCTGTCGGGATTAGATGACGA	
cYFP-FgExosc1-OF	ctgaaacagaaggtcatgaaccacATGGCCGTCGACGACATCCCCTCCG	
cYFP-FgExosc1-OR	CCACTAGCTCCAGCGGCGCGCCGAAAAGGCAGTATTCTAACAAAGCA	
FgExosc1-cYFP-R	cttgcaggccgggcgaagcttGCTAGGCTTTGCGACTTTTCGAGGC	
FgExosc2-nYFP-F	agggaacaaaagctgggtaccCCTTTGCACCTCGAACTT	
FgExosc2-nYFP-R	cgtggcgatggagcgaagcttGTGACCAGCCACAGCAGTTGCTAGA	
FgExosc3-nYFP-F	agggaacaaaagctgggtaccACCCTTGCCGCCGACTTCA	
FgExosc3-nYFP-R	cgtggcgatggagcgaagcttGCTTAGCTCCTTGATGATTTTCCTC	BiFC assay
FgExosc4-nYFP-F	agggaacaaaagctgggtaccAAAGACGCCAACATTTCT	
FgExosc4-nYFP-R	cgtggcgatggagcgaagcttTCCATCCAAATCCATCACCGCGTCA	
FgExosc5-nYFP-F	agggaacaaaagctgggtaccGCCACAAGCATCAAAGCC	
FgExosc5-nYFP-R	cgtggcgatggagcgaagcttTTTCCAGTAGAGATCTTCCGCCACT	
FgExosc6-nYFP-F	agggaacaaaagctgggtaccGGAGACAAGCGAACACGG	
FgExosc6-nYFP-R	cgtggcgatggagcgaagcttGACCGCAAGGATTGATTCGTTGAGA	
FgExosc7-nYFP-F	cgtggcgatggagcgaagcttTGAGAGCTCGACACCCTCCAAACCA	
FgExosc7-nYFP-R	agggaacaaaagctgggtaccTGCTCCGCCGATGTTTCC	
nYFP-FgExosc8-PF	GGGTACCGGGCCCCCCCTCGAGTCCAAGCAACGGGTAATC	
nYFP-FgExosc8-PR	TCCTCGCCCTTGCTCACCATTTCTTTTTGAGACGCAGAATTGGAA	
nYFP-FgExosc8-OF	ATGGCCcgctccatcgccacgATGGCGTCAACAACAGGACTTACTC	

nYFP-FgExosc8-OR	CCCCCGGGCTGCAGGAATTCCCACTCAGCCCTTTATGC	
FgExosc9-nYFP-F	agggaacaaaagctgggtaccCTTTGGTCACTCATTGGTCGTT	
FgExosc9-nYFP-R	cgtggcgatggagcgaagcttCCTATCGTTCTCGGCCGTCAACTCT	
FgExosc10-nYFP-F	ATGGCCcgctccatcgccacgatgGAGAAGTCCCAGGATTTCAAGT	
FgExosc10-nYFP-R	CCCCCGGGCTGCAGGAATTCTTGTAGTCTAAGCCCGTAA	
FgExosc11-nYFP-F	agggaacaaaagctgggtaccTCCCAATCGTCCAGTTTA	
FgExosc11-nYFP-R	cgtggcgatggagcgaagcttTGCTTCGATCAGCTCCATCTTGACA	BiFC assay
FgExoscA-nYFP-F	agggaacaaaagctgggtaccTGCTCCGCCGATGTTTCC	
FgExoscA-nYFP-R	cgtggcgatggagcgaagcttGTTTTTGGACTTGCTTTTCT	
FgExoscA-cYFP-F	agggaacaaaagctgggtaccTGCTCCGCCGATGTTTCC	
FgExoscA--cYFP-R	cttgcaggccgggcgaagcttGTTTTTGGACTTGCTTTTCT	
FgEXOSC2 QF	CAACTATGCGTGAGATTGC	
FgEXOSC2 QR	GGTACAGATCATCGTCAGT	
*FgEXOSC3*QR	CCAGAACTTGCCATTACG	
FgEXOSC4 QF	CTACTCGCTGCTCTAATCA	
FgEXOSC4 QR	TCCTCCTGGTTGTTCAAG	
FgEXOSC5 QF	CCGCCATTCCTCTAAAGA	
FgEXOSC5 QR	TGTCCAAGTTGAAGAACCT	
FgEXOSC6 QF	CGGAGCATTCACAGATTC	
FgEXOSC6 QR	GACCGCAAGGATTGATTC	qRT-PCR analysis
FgEXOSC7 QF	TCACTGGAGACTATACCTTG	
FgEXOSC7 QR	CTCCCAATCATCGTCAAAC	
FgEXOSC8 QF	GTCTGTTGTGGCTTACTG	
FgEXOSC8 QR	CTTGATTCTTTGCGGGAG	
FgEXOSC9 QF	ACCGAATCACTATGTCTCAT	
FgEXOSC9 QR	GCTTGAATCAGGCTTACG	
FgEXOSC10 QF	GCTTACTGCCTCTTAATGC	
FgEXOSC10 QR	GACTGTGACCTCTCCATC	

FgEXOSC11 QF	CAATGCGTTGACTCTAAGG	
FgEXOSC11 QR	GGTGAATGGCTTCGTAATC	
Fgβ-Tubulin QF	TCTGACTTCAGGAATGGTCGTTAC	
gβ-Tubulin QR	AGCGGTCTGGATGTTGTTGG	qRT-PCR analysis
FgTRI1 QF	TCCAGACTACGAAGTGCTA	
FgTRI1 QR	TCATCCTGTACCAATTCCAAT	
FgTRI4 QF	ACCAGGTCCTCAGTCTTG	
FgTRI4 QR	TCGTTGTGCTTGCCATAG	qRT-PCR analysis
FgTRI5 QF	TGAGGGATGTTGGATTGAGCAGTAC	
FgTRI5 QR	TGCTTCCGCTCATCAAACAGGT	
FgTRI6 QF	GCTACTCAGAATGCCCTCAG	
FgTRI6 QR	CGCATGTTATCCACCCTGCTA	
FgTRI10 QF	GCTGTAACTGTCCCCAGCAT	qRT-PCR analysis
FgTRI10 QR	GTGAAGTTGCGACCGTACTC	
FgTRI12 QF	ATGATGATTGAGGATATGTTGT	
